# Mitoxantrone and Analogues Bind and Stabilize i-Motif Forming DNA Sequences

**DOI:** 10.1038/srep39456

**Published:** 2016-12-22

**Authors:** Elisé P. Wright, Henry A. Day, Ali M. Ibrahim, Jeethendra Kumar, Leo J. E. Boswell, Camille Huguin, Clare E. M. Stevenson, Klaus Pors, Zoë A. E. Waller

**Affiliations:** 1 School of Pharmacy, University of East Anglia, Norwich Research Park, Norwich, NR4 7TJ, UK; 2 Institute of Cancer Therapeutics, Faculty of Life Sciences, University of Bradford, Bradford, West Yorkshire, BD7 1DP, UK; 3 Department of Biological Chemistry, John Innes Centre, Norwich Research Park, Norwich, NR4 7UH, UK; 4 Centre for Molecular and Structural Biochemistry, University of East Anglia, Norwich Research Park, Norwich, NR4 7TJ, UK

## Abstract

There are hundreds of ligands which can interact with G-quadruplex DNA, yet very few which target i-motif. To appreciate an understanding between the dynamics between these structures and how they can be affected by intervention with small molecule ligands, more i-motif binding compounds are required. Herein we describe how the drug **mitoxantrone** can bind, induce folding of and stabilise i-motif forming DNA sequences, even at physiological pH. Additionally, **mitoxantrone** was found to bind i-motif forming sequences preferentially over double helical DNA. We also describe the stabilisation properties of analogues of **mitoxantrone**. This offers a new family of ligands with potential for use in experiments into the structure and function of i-motif forming DNA sequences.

i-Motifs are quadruplex DNA secondary structures formed from cytosine-rich sequences and stabilised by intercalated, hemi-protonated cytosine-cytosine base pairs^1^. Putative i-motif forming sequences occur throughout the genome, typically opposing regions which can form G-quadruplexes; they are particularly enriched in gene promoters^2^,^3^,^4^, suggestive of their involvement in gene transcription. Evidence of the effects and potential roles of i-motifs in biology are limited by previous assumptions that i-motifs always require acidic conditions to form and the subsequent lack of chemical tools and ligands which can be used in their study^5^. Nevertheless, stabilisation of the human telomeric i-motif with single walled carbon nanotubes has been shown to inhibit telomerase activity and interfere with telomere biology^6^,^7^. Furthermore, stabilisation of a promoter i-motif in the bcl-2 oncogene by steroidal-based compounds resulted in a subsequent increase in gene expression^8^,^9^. In contrast to the hundreds of G-quadruplex binding ligands, there are very few i-motif binding compounds reported in the literature^5^. To improve the development of understanding into the potential roles of i-motif structures in the genome, a more diverse tool-box of potential compounds is required. Herein we describe a new family of i-motif binding ligands which can preferentially stabilise i-motif forming DNA sequences, even at physiological pH.

## Results and Discussion

**Figure 1:**
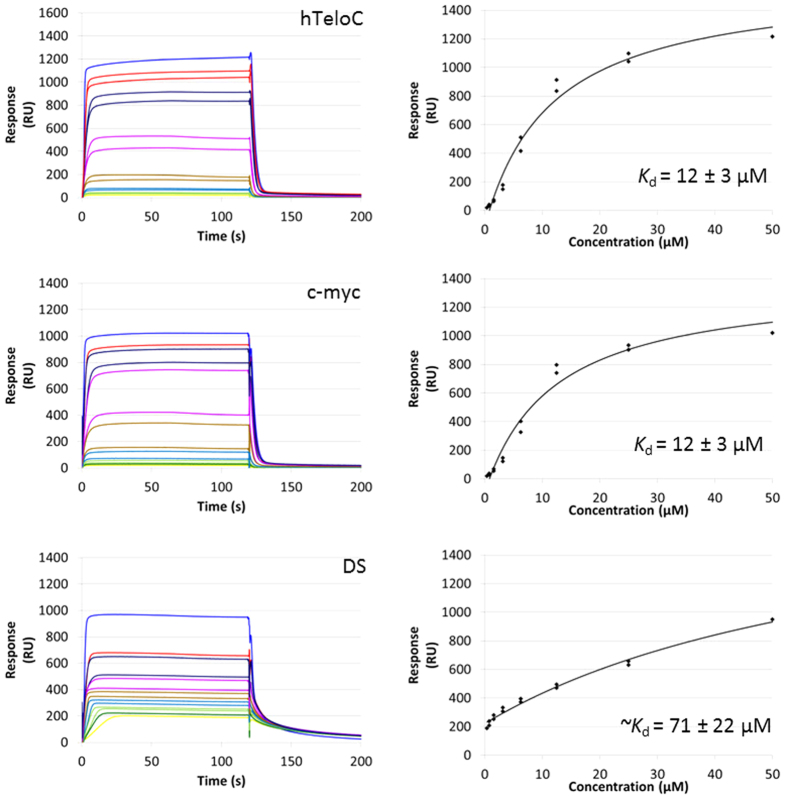
Example sensorgrams (left) and fittings (right) for mitoxantrone with hTeloC_biotin_ (top) c-myc_biotin_ (middle) and DS_biotin_ (bottom) in pH 5.5 10 mM sodium cacodylate supplemented with 100 mM NaCl, 0.05% tween-20 and 5% DMSO. Sensorgrams are double referenced and solvent corrected.

Given the scant literature surrounding i-motif binding compounds, we decided to use a screening strategy to identify any potential leads. We used a medium throughput Fӧrster resonance energy transfer (FRET)-based DNA melting screen^10^ of a 960 compound library from MicroSource against the i-motif forming sequence from the human telomere (hTeloC_FRET_, 5′-FAM-[TAA-CCC-TAA-CCC-TAA-CCC-TAA-CCC]-TAMRA-3′) at pH 5.5, where this sequence is mainly in a folded conformation. The MicroSource library houses a wide range of known drugs, natural products and biologically active compounds. From the initial screen (Fig. S1) we identified **mitoxantrone** as a suitable lead compound from which to start further binding studies. **Mitoxantrone** is a known type 2 topoisomerase inhibitor^11^ and a drug used in the treatment of leukemia, non-Hodgkin’s lymphoma, metastatic breast cancer^12^ and to slow the progression of multiple sclerosis^13^. Anthraquinone compounds similar to **mitoxantrone**, have also been found to inhibit telomerase activity by stabilisation of the G-quadruplex^14^,^15^,^16^. Supporting circular dichroism (CD) experiments indicated that **mitoxantrone** interacts with i-motif DNA (Fig. S9) so we used surface plasmon resonance (SPR) to measure equilibrium binding between **mitoxantrone** and i-motif DNA. SPR experiments were performed using three different immobilised DNA targets: hTeloC_Biotin_ (5′-biotin-[TAA-CCC-TAA-CCC-TAA-CCC-TAA-CCC]-3′), c-myc_Biotin_ (5′-biotin-[CCT-TCC-CCA-CCC-TCC-CCA-CCC-TCC-CCA]-3′ and also double stranded DNA (DS_biotin_) for comparison, which comprised the ODN d(biotin-[GGC-ATA-GTG-CGT-GGG-CGT-TAG-C]) hybridized with its complementary strand. Example sensorgrams and fittings are given in Fig. 1.

The SPR results showed that **mitoxantrone** has moderate affinity for the i-motif at pH 5.5, with dissociation constants in the low micromolar range. The affinity for hTeloC and c-myc were found to be the same within error (*K*_d_ = 12 ± 3 and 12 ± 3 μM for hTeloC and c-myc respectively). However, the affinity for double stranded DNA was found to be significantly weaker and because of this could not be as accurately determined using the same range of concentrations. Nevertheless, the dissociation constant was found to be approximately five times lower than i-motif (approximate *K*_d_ = 71 ± 22 μM). This indicates a definite preference towards i-motif structures in the equilibrium binding studies. These affinities are of a similar magnitude to existing i-motif ligands such as the phenanthrolines (4–8 μM at pH 5.5)^17^ but slightly better than the previously described terbium complexes (22–30 μM at pH 5.5)^18^ and the cationic porphyrin **TmPyP4** (45 μM at pH 4.5)^19^.

To investigate how **mitoxantrone** interacts with different types of DNA, further FRET-based melting experiments were performed across a wider range of conditions and types of DNA secondary structure. DNA melting experiments provides a measure of the ligand-induced stabilisation of a folded structure. FRET melting experiments have advantages over other DNA-based melting experiments in that screening of a large range of conditions/ligand concentrations is possible; lower concentrations are required and it avoids the problem that many ligands absorb in the same region as DNA, which may interfere with the absorbance or ellipticity signal resulting from complex dissociation^20^. However, the technique can give rise to experimental artefacts from inherently fluorescent ligands and compounds which interact with the fluorophores rather than the DNA itself^21^. The fluorophores can also alter the folding properties of the DNA^22^. The technique is used widely for assessment of DNA-ligand interactions and we chose to mimic the conditions used by others studying i-motif ligands^6^,^23^. The FRET melting experiments were performed using **mitoxantrone** and a range of dual-labelled oligonucleotides: i-motif forming sequences from the human telomere (hTeloC_FRET_) and the c-myc oncogene (c-myc_FRET_, 5′-FAM-[TCC-CCA-CCT-TCC-CCA-CCC-TCC-CCA-CCC-TCC-CCA]-TAMRA-3′); G-quadruplex forming sequence from the human telomere (hTeloG_FRET_, 5′-FAM-[GGG-TTA-GGG-TTA-GGG-TTA-GGG]-TAMRA-3′) and a duplex forming sequence (DS_FRET_, 5′-FAM-[TAT-AGC-TAT-A-HEG(18)-TAT-AGC-TAT-A]-TAMRA-3′). To support the previous experiments with i-motif, which were performed at acidic pH (pH 5.5), we also performed experiments with i-motif forming sequences at their respective transitional pHs (pH_T_: 6 for hTeloC and 6.6 for c-myc)^24^,^25^, the pH at which the DNA structure is 50% folded. Experiments at pH_T_ allow studies at a higher pH, where part of the population in solution remains folded into i-motif, but is closer to physiological pH. Given the *K*_d_ of **mitoxantrone** with i-motif was 12 μM, initially 10 μM ligand concentration was used across all the experiments and the buffer and cation concentration was also kept constant (10 mM sodium cacodylate, 100 mM NaCl). On addition of 10 μM **mitoxantrone** to the DNA, the change in melting temperature (∆*T*_m_) for i-motif DNA at pH 5.5 was high (hTeloC ∆*T*_m_ = +34 °C and c-myc = +31 °C). However, when the experiments were performed at the transitional pHs, the stabilisation was even higher for both hTeloC (∆*T*_m_ = +42 °C at pH 6) and c-myc (∆*T*_m_ = +38 °C at pH 6.6). **Mitoxantrone** was also found stabilise double helical DNA, but to a much lesser degree (∆*T*_m_ = +6.4 °C), which is consistent with the *K*_d_ values obtained by SPR. It is unsurprising that **mitoxantrone** was also found to stabilise the G-quadruplex forming sequence from the human telomere (hTeloG, ∆*T*_m_ = +16 °C), but again, the stabilisation was not as high as that for the i-motif forming sequences at the same concentration.

**Figure 2:**
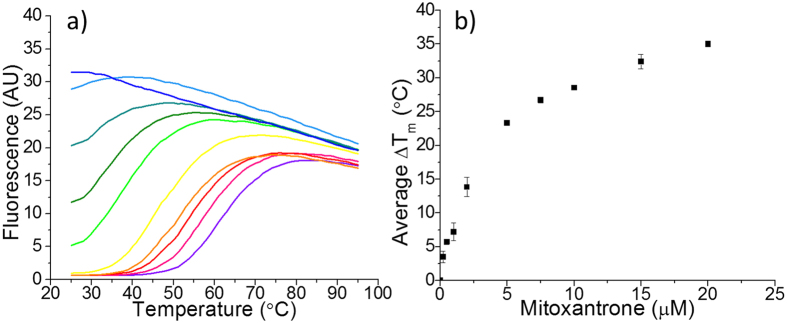
(**a**) Example FRET melting curves for hTeloC (200 nM) with 0, 0.2, 0.5, 1, 2, 5, 7.5, 10 and 20 μM mitoxantrone in pH 7.4 10 mM sodium cacodylate and 100 mM NaCl. (**b**) Plots of change in DNA melting temperature against concentration of mitoxantrone for hTeloC in 10 mM sodium cacodylate at pH 7.4 with 100 mM, the error bars represent the standard deviation from three experiments.

Although at physiological pH the i-motif forming sequences from the human telomere and c-myc reside in a predominantly unfolded conformation^24^,^25^, we were encouraged by the positive results in the experiments performed at transitional pH. Performing analogous melting experiments at pH 7.4 gave a stabilisation temperatures of +27 °C for hTeloC and +29 °C for c-myc, indicating that **mitoxantrone** can induce folding of hTeloC and c-myc in the absence of the acidic conditions typically required for these particular sequences. Further experiments across a wider concentration range with hTeloC shows a dose-dependent increase in folded DNA structure (Fig. 2a). At pH 7.4, hTeloC is unfolded at 25 °C, indicated in the FRET experiments by high fluorescence signal across the whole temperature range (*T*_m_ < 25 °C). At the lowest concentration of mitoxantrone (0.2 μM) there is a slight reduction in fluorescence signal at the start of the melting experiment (25 °C), indicating part of the DNA population is folded. This reduction increases in a concentration-dependent manner until 5 μM, where the fluorescence is fully quenched, indicating predominantly folded populations at 25 °C. Higher concentrations of **mitoxantrone** gave further increases in melting temperature. Stabilisation temperatures were calculated assuming a *T*_m_ of 25 °C in the absence of any ligand, i.e. the minimum possible *T*_m_ under the conditions used in this experiment. A concentration versus *T*_m_ plot is shown in Fig. 2b. Analogous experiments with c-myc showed similar behavior (Fig. S2). Further example melting curves and concentration versus *T*_m_ plots for all the DNA structures (and conditions) are also provided in the SI.

**Figure 3:**
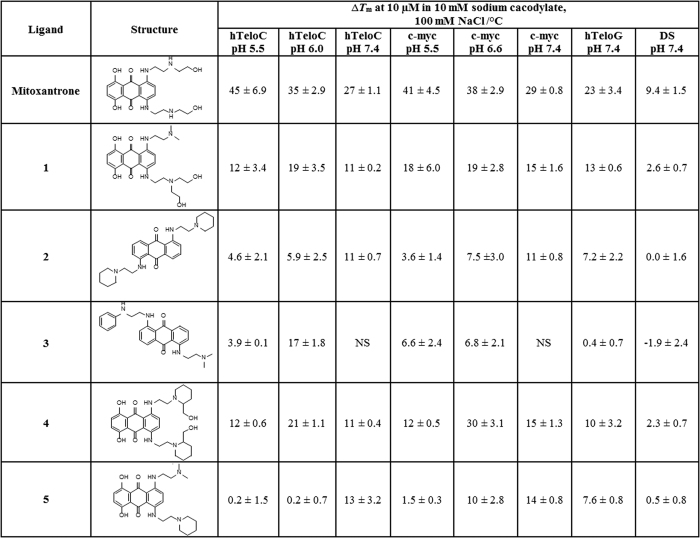
Structures of the anthraquinones and stabilisation potentials (∆*T*_m_) determined by FRET melting. Errors represent the standard deviation from three independent experiments. NS indicates where the ligand did not induce stabilisation of the DNA.

Although carboxyl modified single walled carbon nanotubes have been shown to stabilise i-motif forming DNA sequences at neutral pH^6^, to the best of our knowledge, this is not yet documented in the literature for small molecule ligands. Nevertheless, **mitoxantrone** still has some interaction with double helical DNA in both the FRET and SPR experiments. To see whether it was possible to gain higher specificity for i-motif over double helical DNA, we decided to investigate the stabilisation capabilities of analogue compounds. Previous studies using analogues of **mitoxantrone** allowed easy access to a mixture of both known and novel structures^26^,^27^,^28^,^29^,^30^. An initial screen of 25 analogues was performed (Fig. S10); selecting ligands which stabilised i-motif, but not double helical DNA at the same concentration, gave rise to a subset of ligands **1**–**5** which were repeated and are reported in Fig. 3.

The advantage that the analogue compounds (**1**–**5**) have over **mitoxantrone** is their reduced ability to stabilise double helical DNA, with Δ*T*_m_ values between −3 and +2 °C. Furthermore, all of the analogues reported in Fig. 2 also have reduced stabilization potential for the human telomeric G-quadruplex (hTeloG) with Δ*T*_m_ values between +0.4 and +13 °C, compared to +16 °C for **mitoxantrone**. For example, **3** does not stabilise G-quadruplex DNA at all, with a Δ*T*_m_ of +0.4 °C for G-quadruplex. The same ligand can also stabilise hTeloC well (Δ*T*_m_ = +17 °C at pH 6), this indicates some preference for i-motif. However, unfortunately this analogue did not induce stabilisation of the i-motif forming sequences at pH 7.4.

Further SPR experiments were performed at pH 5.5 to give an indication of the binding properties of the analogue compounds against i-motif and double stranded DNA. These revealed that ligands **1**–**5** did not bind i-motif as well as **mitoxantrone** at this pH, which is consistent with the overall reduced stabilisation profiles measured at pH 5.5. For example the next best compound, ligand **2**, had lower affinity for all the structures; the dissociation constants for hTeloC and c-myc were found to be the same within error (*K*_d_ = 31 ± 5.1 and 34 ± 6.7 μM for hTeloC and c-myc respectively) and the affinity for double stranded DNA was again found to be approximately five times lower than i-motif (approximate *K*_d_ = 181 μM) at pH 5.5.

The FRET melting and SPR results indicate that **mitoxantrone** and the analogues **1**–**5** could be useful in different studies into i-motif structure and function, with the potential to choose from subtly different ligands which have different pH dependent stabilisation profiles. Although the analogues in this study were not found to have improved selectivity beyond that of the parent compound **mitoxantrone**, this does not rule out the possibility that further selectivity for i-motif forming sequences over other DNA secondary structures may be obtained with the creation of further analogues based around these scaffolds.

When compared to other small molecule i-motif binding ligands in the literature, **mitoxantrone** and the analogues described herein offer preferential stabilisation of i-motif forming sequences and some even at neutral pH. The cholestane derivative **IMC-48** (**NSC 138948**), which binds the BCL-2 i-motif, only has a Δ*T*_m_ of +1 °C^9^ and the previously reported terbium^18^ and ruthenium^31^ complexes did not stabilise i-motif structures at all. **BisA**, which has been shown to interact with the human telomeric i-motif, has a Δ*T*_m_ of +33 °C, but this was at pH 6.8^23^. Overall, **mitoxantrone** and analogues offer a small-molecule scaffold which can both bind i-motif DNA and stabilize i-motif forming sequences better than double helical DNA.

## Conclusions

Herein we have described for the first time that **mitoxantrone** is able to both bind and stabilise i-motif forming DNA sequences preferentially over double helical DNA. Furthermore, to the best of our knowledge, this is the first example of a small molecule which can stabilise i-motif forming DNA sequences at neutral pH, thus the compounds described here have potential for use in the study of i-motif DNA structure and function.

## Methods

### General Experimental

All the oligonucleotides (ODNs) and their fluorescent conjugates were purchased from Eurogentec and were HPLC purified. Solid DNA samples were initially dissolved as a stock solution in MilliQ water (100 μM for labelled and 1 mM for unlabeled ODNs); further dilutions were carried out in the respective sodium cacodylate buffer. Annealed samples were thermally annealed in a heat block at 95 °C for 5 minutes and cooled slowly to room temperature overnight. Non-annealed samples had the DNA diluted into the respective buffer and were used immediately. The Gen-Plus library from Microsource Discovery Systems Inc. consisting of 960 drug standards with approval in Europe, Japan or the USA, supplied as 10 mM solutions in DMSO which were diluted to 1 mM in 96 well plates. **Mitoxantrone** was purchased as the di-hydrochloride salt from Molekula. Stock solutions of ligands at 10 mM were made in purified water and/or DMSO and were stored at −20 °C, subsequent dilutions were made in the appropriate buffer.

For the synthesis of the novel anthraquinones (anthracene-9,10-diones), all chemicals were obtained from Aldrich (Poole, Dorset), Lancaster (Morecambe, Lancashire) and VWR (Poole, Dorset). All other solvents were supplied by VWR. Reagents were used as received. Flash chromatography was carried out on silica gel [Merck 9385 Kieselgel 60 (230–400 ASTM) supplied by VWR]. Analytical TLC was carried out on 0.25 mm thick aluminium plates precoated with Merck Kieselgel F254 silica gel (VWR) and visualised by UV and aqueous alkaline potassium permanganate solution. NMR spectra were recorded on Jeol GX270, Jeol AM600 or Bruker DPX400 spectrometers. Purity of all compounds are ≥95% as measured by HPLC (see SI).

### FRET Melting Screen

The initial DNA melting screen was performed using a fluorescence resonance energy transfer (FRET) DNA melting based assay. The labelled oligonucleotide hTeloC_FRET_ (5′-FAM-d[TAA-CCC-TAA-CCC-TAA-CCC-TAA-CCC]-TAMRA-3′; donor fluorophore FAM is 6-carboxyfluorescein; acceptor fluorophore TAMRA is 6-carboxytetramethyl-rhodamine) was prepared as a 220 nM solution in 10 mM sodium cacodylate buffer at pH 5.5 with 100 mM sodium chloride and then thermally annealed. Strip-tubes (QIAgen) were prepared by aliquoting 18 μL of the annealed DNA, followed by 2 μL of 1 mM compound library solutions. Control samples for each run were prepared with the same quantity of DMSO with the DNA in buffer. Fluorescence melting curves were determined in a QIAgen Rotor-Gene Q-series PCR machine, using a total reaction volume of 20 μL. Samples were held at 25 °C for 5 minutes then ramped to 95 °C at increments of 1 °C, holding the temperature at each step for 1 minute. Measurements were made with excitation at 470 nm and detection at 510 nm. DNA melting points were determined using the first derivative of the melting curve, any experiments where the inflection point was not able to be determined (i.e. the transition does not occur before the end of the experiment) were defined to have a *T*_m_ of >95 °C. Initial Hits against hTeloC were repeated in 10 mM sodium cacodylate buffer at pH 5.5 at both high and low salt concentrations (100 mM and 5 mM NaCl). Further FRET melting experiments were performed using 200 nM DNA in 10 mM sodium cacodylate supplemented with 100 mM NaCl with the ligand **Mitoxantrone** (or respective analogue) added. hTeloC_FRET_ and c-Myc_FRET_ (5′-FAM-d[TCC-CCA-CCT-TCC-CCA-CCC-TCC-CCA-CCC-TCC-CCA]-TAMRA-3′) were tested at their respective transitional pHs (6.0 and 6.6), acidic (pH 5.5) and physiological (pH 7.4). hTeloG_FRET_ (5′-FAM-d[GGG-TTA-GGG-TTA-GGG-TTA-GGG]-TAMRA-3′) and DS_FRET_ FAM-d(TAT-AGC-TAT-A-HEG(18)-TAT-AGC-TAT-A)-TAMRA-3′) were measured at pH 7.4. Final analysis of the data was carried out using QIAgen Rotor-Gene Q-series software and Origin or Excel.

### Circular Dichroism

Circular dichroism (CD) spectra were recorded on a Jasco J-810 spectropolarimeter using a 1 mm path length quartz cuvette. Human telomeric i-motif (hTeloC, 5′d[TAA-CCC-TAA-CCC-TAA-CCC-TAA-CCC]-3′) was diluted in a buffer containing 10 mM sodium cacodylate and 100 mM NaCl at pH 5.5, to achieve a total volume of 200 μL. The scans were performed at 20 °C over a wavelength range of 220–400 nm with a scanning speed of 200 nm/min, a response time of 1 s, 0.5 nm pitch and 2 nm bandwidth. A blank sample containing only buffer (and, where necessary ligand) was treated in the same manner and subtracted from the collected data. Solutions of mitoxantrone were added in small aliquots to the desired equivalent proportions using a pipette. The CD spectra represent an average of three scans and are zero corrected at 320 nm. Final analysis and processing of the data was performed using Origin.

### Surface Plasmon Resonance

SPR experiments were performed using a GE Healthcare Biacore T200 instrument with a series S streptavidin (SA) coated chip. For immobilization all DNA samples were biotinylated. hTeloC_Biotin_, (5′-biotin-d[TAA-CCC-TAA-CCC-TAA-CCC-TAA-CCC]-3′) c-Myc_Biotin_ (5′-biotin-d[CCT-TCC-CCA-CCC-TCC-CCA-CCC-TCC-CCA]-3′) sequences were diluted to 1 μM in running buffer (10 mM sodium cacodylate (pH 5.5), 100 mM NaCl and 0.05% Tween-20) and the Double stranded DNA DS_Biotin_ (5′-biotin-d[GGC-ATA-GTG-CGT-GGG-CGT-TAG-C]-3′) was annealed with its complimentary strand at 1 μM in running buffer. For immobilization, the chip was first conditioned with three 60 s washes of 1 M NaCl and 50 mM NaOH at a flow rate of 10 μL min^−1^ to remove any unconjugated streptavidin. The biotinylated oligonucleotides were then injected over flow cells 2 (hTeloC_Biotin_,761.7 RU), 3 (c-Myc_Biotin_, 664.0 RU) and 4 (DS_Biotin_+comp, 568.1 RU) with flow cell 1 left blank.

For affinity measurements, the running buffer was identical but had the addition of 5% DMSO. Ligand stocks (10 mM in DMSO) were serially diluted with buffer without DMSO to give concentrations of 100, 50, 25, 12.5, 6.25, 3.125, 1.56, 0.78, 0.39 and 0 μM in a final composition the same as the running buffer (10 mM sodium cacodylate (pH 5.5), 100 mM NaCl, 0.05% Tween-20 and 5% DMSO). It was crucial that all concentrations of ligand contained 5% DMSO and in addition solvent correction was performed where 8 solutions with varying amounts of DMSO (4.5–5.8%) were also prepared. The solvent correction samples were run at the start and end of the experiment and every 30 cycles. Binding experiments were performed using the affinity run wizard in the Biacore T200 software at 25 °C and a flow rate of 30 μL min^−1^. Prior to sample injection, 1 startup cycle was performed: blank injections of buffer followed by 2 regeneration injections of 1 M NaCl. Each concentration of ligand was injected for 120 s and the responses in each flow cell were measured. After each injection the chip surface was regenerated by two 60 s injections of 1 M NaCl followed by washing with running buffer for 60 s. Each ligand concentration was repeated with a second injection to ensure reproducibility. The response data was solvent corrected and double referenced by subtracting the startup cycle and injections of buffer only samples. Non-selective binding to the chip surface was accounted for by subtracting the response from the blank flow cell. Resultant sensorgrams were fitted using the average equilibrium response for each concentration and fitted using the affinity fit from the Biacore T200 evaluation software v2.0 assuming a 1:1 binding model.

### Mitoxantrone Analogues

**1** and **4**–**5** were made as previously described^20^,^21^,^22^,^23^,^24^.

#### 1,5-bis((2-(piperidin-1-yl)ethyl)amino)anthracene-9,10-dione (2)

1,5-dichloroanthracene-9,10-dione (100 mg, 0.361 mmol) was stirred in 2-(piperidin-1-yl)ethylamine (1 mL) at 100 °C for 24 h before the solution was poured into cold brine. The precipitated solid was isolated by filtration and the crude compound was purified by flash column chromatography using CH_2_Cl_2_:CH_3_OH (98:2 → 90:10) to yield the title compound **2** (141.3 mg, 85%) as a red solid.

^1^H NMR (400 MHz, CDCl_3_): δ (ppm) 9.68 (t, *J* = 4.72 Hz, 2H, Ar-*NH*), 7.48 (m, 4H, *ArH*), 6.89 (d, *J* = 7,2 2H, *ArH*), 3.38 (q, *J* = 6.2 Hz, 4H, ArNH*CH*_*2*_CH_2_), 2.62 (t, 4H, *J* = 6.8 Hz, ArNHCH_2_*CH*_*2*_), 2.43 (m, 8H, N*CH*_*2*_CH_2_CH_2_), 1.58 (m, 8H, NCH_2_*CH*_*2*_CH_2_), 1.40 (m, 4H, NCH_2_CH_2_*CH*_*2*_); ^13^C NMR (101 MHz, CDCl_3_): δ (ppm) 184.31, 150.17, 135.29, 134.08, 115.34, 113.75, 112.12, 56.49, 53.66, 39.43, 24.91, 23.31; m/z 461 ([M + H]^+^, 47%); HRMS (*m/z*): [M+H]^+^ calcd for C_28_H_36_N_4_O_2_, 461.2911; found, 461.2906.

#### 1-((2-(dimethylamino)ethyl)amino)-5-((2-(phenylamino)ethyl)amino)anthracene-9,10-dione (3)

The method follows that of **2** using **13** (see SI) (25 mg, 0.066 mmol) and *N*,*N*-dimethylethane-1,2-diamine (1 mL). The product **3** was afforded as a red solid (15.6 mg, 55%).

^1^H NMR (400 MHz, CDCl_3_): δ (ppm) 9.76 (t, *J* = 5.0 Hz, 1H, Ar*NH*), 9.70 (t, *J* = 4.4 Hz, 1H, Ar*NH*), 7.53 (d, *J* = 7.6 Hz, 1H, *ArH*), 7.45 (m, 3H, *ArH*), 7.13 (t, *J* = 7.2 Hz, 2H, *ArH*), 6.91 (d, *J* = 7.2 Hz, 2H, *ArH*), 6.67 (t, *J* = 7.2 Hz, 1H, *ArH*), 6.61 (d, *J* = 8.0 Hz, 2H, *ArH*), 3.89 (s_br_, 1H, CH_2_*NH*Ph), 3.53 (q, *J* = 6.0 Hz, 2H, NH*CH*_*2*_CH_2_NHPh), 3.42 (s_br_, 2H, NHCH_2_*CH*_*2*_NHPh), 3.35 (q, *J* = 6.4 Hz, 2H, NH*CH*_*2*_CH_2_N), 2.60 (t, *J* = 6.4 Hz, 2H, NHCH_2_*CH*_*2*_N), 2.28 (s, 6H, N*CH*_*3*_); ^13^C NMR (101 MHz, CDCl_3_): δ (ppm) 185.74, 185.23, 151.34, 151.25, 147.60, 136.37, 136.19, 135.30, 135.26, 129.38, 117.89, 116.54, 116.25, 115.38, 114.87, 113.41, 113.08, 113.05, 58.06, 45.62, 43.11, 42.16, 40.97; m/z 429 ([M+H]^+^, 100%); HRMS (*m/z*): [M+H]^+^ calcd for C_26_H_28_N_4_O_2_, 429.2285; found, 429.2285.

## Additional Information

**How to cite this article:** Wright, E. P. *et al*. Mitoxantrone and Analogues Bind and Stabilize i-Motif Forming DNA Sequences. *Sci. Rep.* **6**, 39456; doi: 10.1038/srep39456 (2016).

**Publisher's note:** Springer Nature remains neutral with regard to jurisdictional claims in published maps and institutional affiliations.

## Supplementary Material

Supplementary Information
