# Supplementary material for: Mitoxantrone and Analogues Bind and Stabilize i-Motif Forming DNA Sequences
Source: Sci Rep. 2016 Dec 22;6:39456. doi: 10.1038/srep39456 (PMC5177923; doi:10.1038/srep39456)
Supplement: Supplementary Information [file srep39456-s1.pdf]

Mitoxantrone and Analogues Bind and Stabilise i-Motif Forming DNA Sequences

Elisé P. Wright,[†] Henry A. Day,[†] Ali M. Ibrahim,[‡] Jeethendra Kumar,[‡] Leo J. E. Boswell,[†] Camille Huguin,[†] Clare E. M. Stevenson,[¥] Klaus Pors[‡*] and Zoë A. E. Waller[†§*]

[†]School of Pharmacy, University of East Anglia, Norwich Research Park, Norwich, NR4 7TJ, UK
[‡]Institute of Cancer Therapeutics, Faculty of Life Sciences, University of Bradford, Bradford, West Yorkshire, BD7 1DP, UK
[¥]Department of Biological Chemistry, John Innes Centre, Norwich Research Park, Norwich, NR4 7UH, UK
[§]Centre for Molecular and Structural Biochemistry, University of East Anglia, Norwich Research Park, Norwich, NR4 7TJ, UK

*Corresponding Authors: z.waller@uea.ac.uk, k.pors1@bradford.ac.uk

SUPPORTING INFORMATION for Scientific Reports

CONTENTS

1. FRET MELTING EXPERIMENTS 2
2. CIRCULAR DICHROISM EXPERIMENTS 6
3. SURFACE PLASMON RESONANCE (SPR) EXPERIMENTS 7
5. ANALOGUE LIBRARY FRET SCREEN 8
6. SYNTHETIC PROTOCOLS AND CHARACTERISATION OF MITOXANTRONE ANALOGUES 11
7. HPLC TRACES 22
8. SUPPORTING INFORMATION REFERENCES 39

1. FRET MELTING EXPERIMENTS

The initial hits from the screen, were vetted to remove known cross-linking agents and were repeated again in both high (100 mM NaCl) and low (5 mM NaCl) salt buffer conditions to give 13 final hits which displayed a ΔT_m of at least 5°C (Fig. S1). Out of these, there were some compounds (eg. P2C5 and P9C11) which precipitated, rather than stabilised DNA so these were avoided. Out of the rest P9H8, **mitoxantrone**, offered the best stabilisation potential in both buffer conditions.

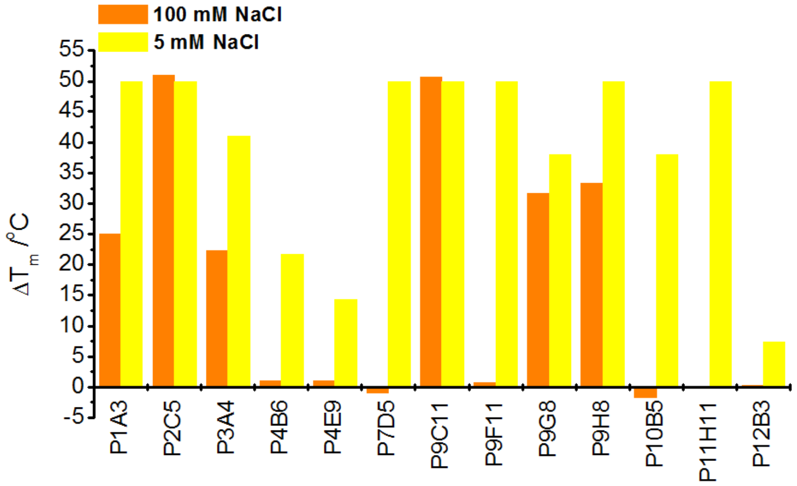

Figure S1: **Comparison of the measured ΔT_m values of the 13 repeated compounds. DNA = 200 nM hTeloC$_{FRET}$, buffer = 10 mM sodium cacodylate and 100 mM or 5 mM NaCl at pH 5.5.**

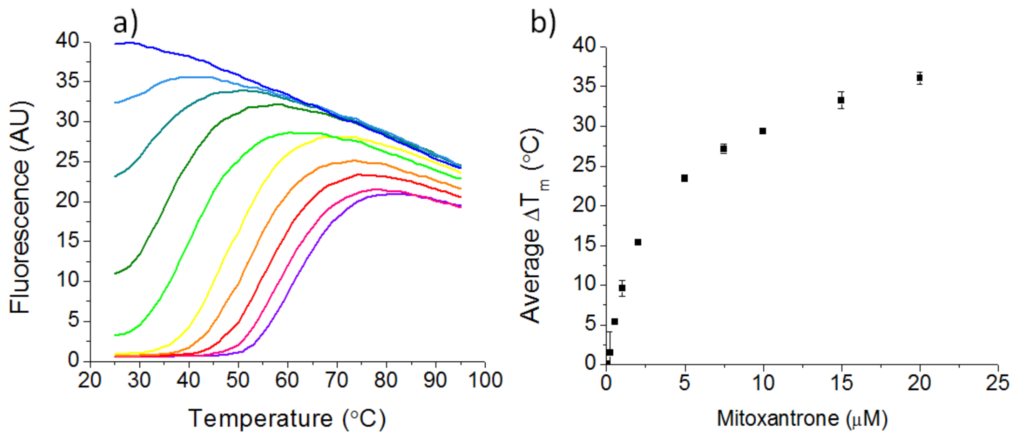

Figure S2: **a) Example FRET melting curves for c-myc$_{FRET}$ (200 nM) with 0, 0.2, 0.5, 1, 2, 5, 7.5, 10 and 10 µM mitoxantrone in pH 7.4 10 mM sodium cacodylate and 100 mM NaCl. b) Plots of change in DNA melting temperature against concentration of mitoxantrone for c-myc in 10 mM sodium cacodylate at pH 7.4 with 100 mM, the error bars represent the standard deviation from three experiments.**

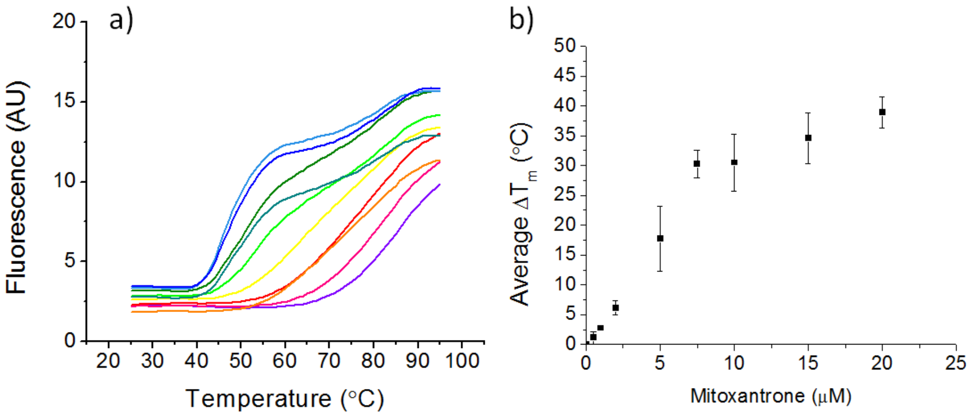

Figure S3: **a) Example FRET melting curves for c-myc$_{FRET}$ (200 nM) with 0, 0.2, 0.5, 1, 2, 5, 7.5, 10 and 10 µM mitoxantrone in pH 5.5 10 mM sodium cacodylate and 100 mM NaCl. b) Plots of change in DNA melting temperature against concentration of mitoxantrone for c-myc in 10 mM sodium cacodylate at pH 5.5 with 100 mM, the error bars represent the standard deviation from three experiments.**

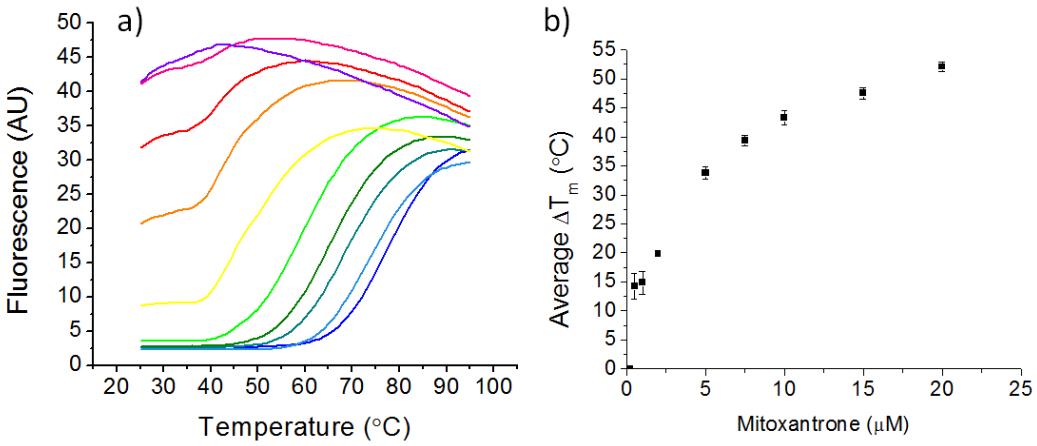

Figure S4: **a) Example FRET melting curves for c-myc$_{FRET}$ (200 nM) with 0, 0.2, 0.5, 1, 2, 5, 7.5, 10 and 10 µM mitoxantrone in pH 6.6 10 mM sodium cacodylate and 100 mM NaCl. b) Plots of change in DNA melting temperature against concentration of mitoxantrone for c-myc in 10 mM sodium cacodylate at pH 6.6 with 100 mM, the error bars represent the standard deviation from three experiments.**

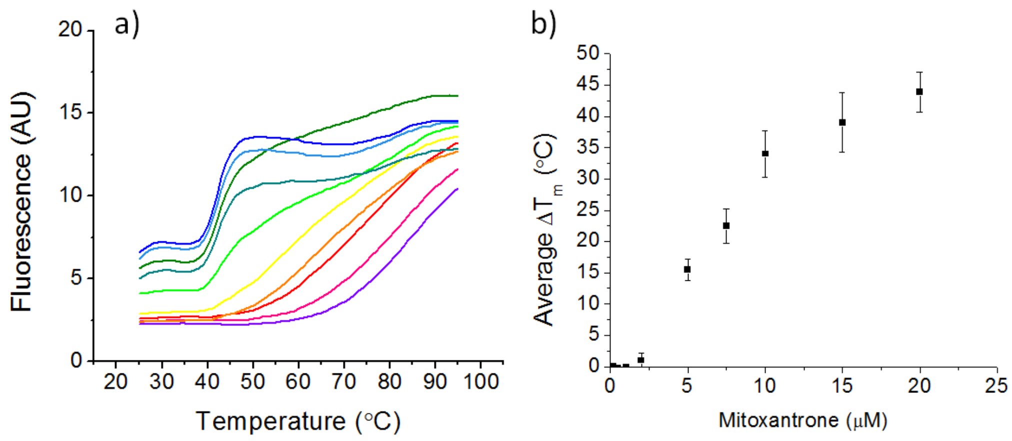

Figure S5: **a) Example FRET melting curves for hTeloC$_{FRET}$ (200 nM) with 0, 0.2, 0.5, 1, 2, 5, 7.5, 10 and 10 μM mitoxantrone in pH 5.5 10 mM sodium cacodylate and 100 mM NaCl. b) Plots of change in DNA melting temperature against concentration of mitoxantrone for hTeloC in 10 mM sodium cacodylate at pH 5.5 with 100 mM, the error bars represent the standard deviation from three experiments.**

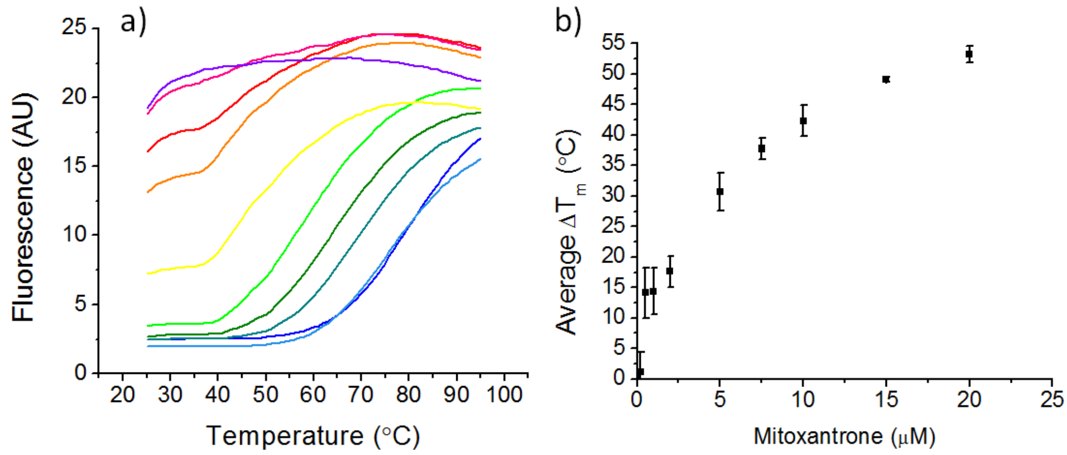

Figure S6: **a) Example FRET melting curves for hTeloC$_{FRET}$ (200 nM) with 0, 0.2, 0.5, 1, 2, 5, 7.5, 10 and 10 μM mitoxantrone in pH 6.0 10 mM sodium cacodylate and 100 mM NaCl. b) Plots of change in DNA melting temperature against concentration of mitoxantrone for hTeloC in 10 mM sodium cacodylate at pH 6.0 with 100 mM, the error bars represent the standard deviation from three experiments.**

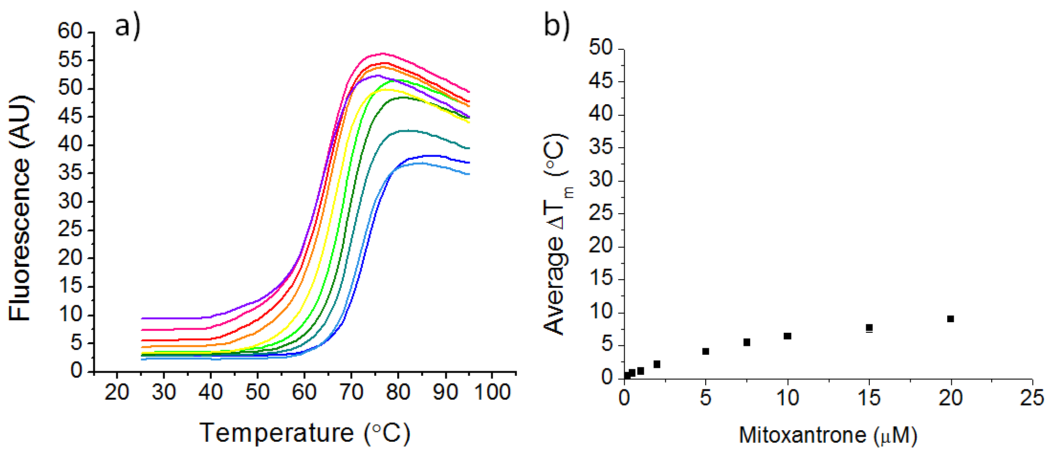

Figure S7: **a) Example FRET melting curves for DS$_{FRET}$ (200 nM) with 0, 0.2, 0.5, 1, 2, 5, 7.5, 10 and 10 µM mitoxantrone in pH 7.4 10 mM sodium cacodylate and 100 mM NaCl. b) Plots of change in DNA melting temperature against concentration of mitoxantrone for DS in 10 mM sodium cacodylate at pH 7.4 with 100 mM, the error bars represent the standard deviation from three experiments.**

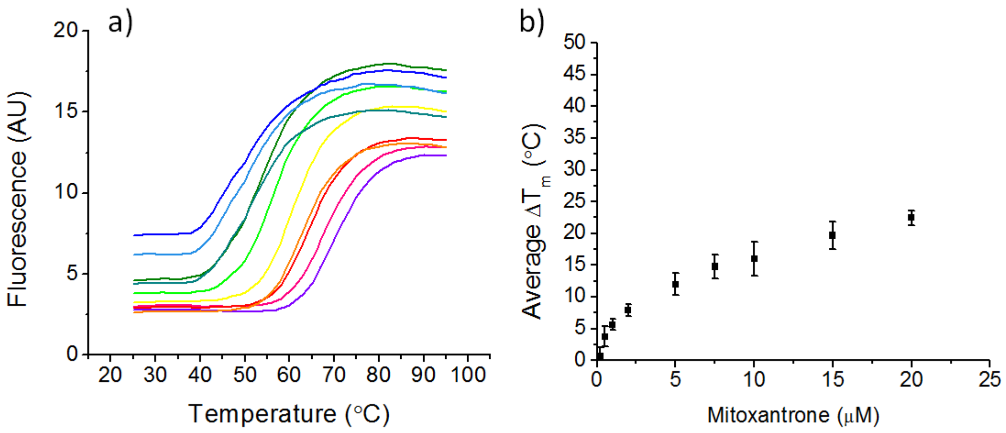

Figure S8: **a) Example FRET melting curves for hTeloG$_{FRET}$ (200 nM) with 0, 0.2, 0.5, 1, 2, 5, 7.5, 10 and 10 µM mitoxantrone in pH 7.4 10 mM sodium cacodylate and 100 mM NaCl. b) Plots of change in DNA melting temperature against concentration of mitoxantrone for DS in 10 mM sodium cacodylate at pH 7.4 with 100 mM, the error bars represent the standard deviation from three experiments.**

2. CIRCULAR DICHROISM EXPERIMENTS

The CD spectrum of hTeloC in 10 mM sodium cacodylate and 100 mM NaCl at pH 5.5 has a negative signal at 255 nm and a positive signal at 288 nm, consistent with i-motif structure.[1] Titration of 10 µM **mitoxantrone** into the i-motif sample did not alter the signal, but further additions of ligand resulted in a decrease in the positive signal at 288 nm and a shift towards longer wavelengths (Fig. S9). The resulting signals are not consistent with any known DNA structure and are most likely to be the result of an induced CD signal, which indicates binding between the ligand and the DNA.[2]

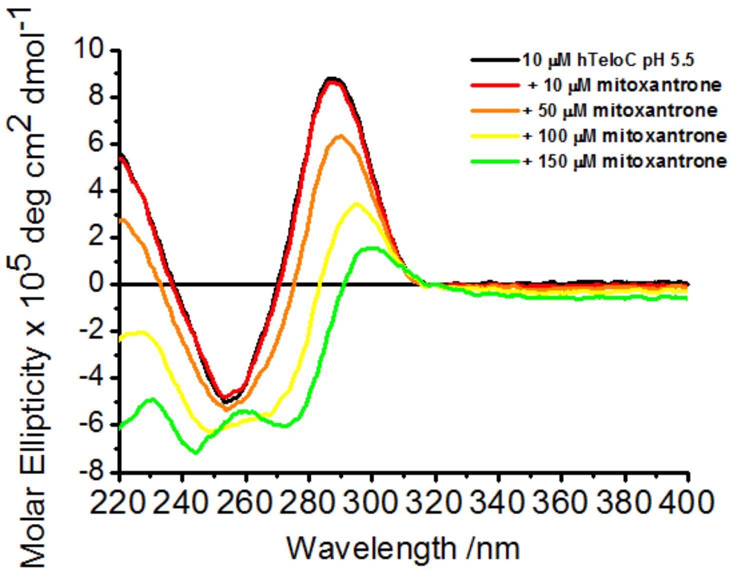

Figure S9: **Example circular dichroism experiments of hTeloC (10 µM in 10 mM sodium cacodylate and 100 mM NaCl at pH 5.5) with 0-150 µM mitoxantrone.**

3. SURFACE PLASMON RESONANCE (SPR) EXPERIMENTS

Table S1: Dissociation Constants (K_d, μM) Determined by SPR in pH 5.5 10 mM sodium cacodylate, 100 mM NaCl, 0.05% Tween-20 and 5% DMSO.

Compound	Dissociation constant, K_d (μM)		
	**hTeloC**	**c-myc**	**DS**
**Mitoxantrone**	12 ± 3	12 ± 3	71 ± 22
**1**	31 ± 5	34 ± 7	181 ± 90
**2**	99 ± 30	251 ± 70	15 ± 2
**3**	NSB	NSB	NSB
**4**	80 ± 15	92 ± 20	34 ± 12
**5**	36 ± 10	42 ± 12	33 ± 11
**6**	NSB	NSB	8.3 ± 1.4

4.

5. ANALOGUE LIBRARY FRET SCREEN

The screening experiments were performed once (Fig. S10). Key ligands which did not show remarkable interaction with ds DNA were repeated another two times and these are given in the main manuscript.

Figure S10. **Numbering and structures of the anthraquinones, source or synthesis route, and stabilisation potentials (ΔT_m) determined by FRET melting.**

Compound		Source/ Route	ΔT_m at 10 μM [ligand] with 0.2 μM [DNA]					
			hTeloC pH 5.5	hTeloC pH 6.0	cMycC pH 5.5	cMycC pH 6.6	hTeloG pH 7.4	DS pH 7.4
**6**	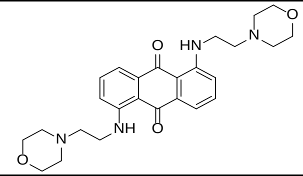	Fig. S11	1.9	4.3	0.2	-1	1	-1
**7**	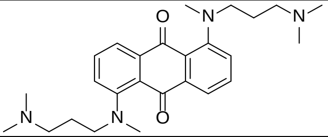	Fig. S11	-1.3	0	-1.3	-8	3.2	-2.9
**8**	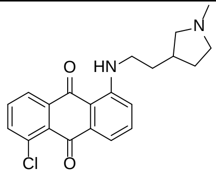	Fig. S11	0.5	2.8	1	8.7	8.5	1.9
**9**	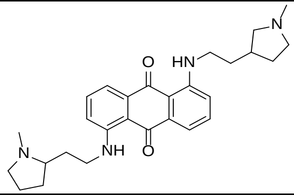	Fig. S11	22.1	23.3	21.5	20.6	19	9.1
**10**	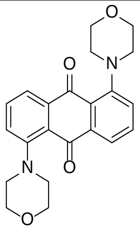	Fig. S11	0.1	-0.6	-2	-16.5	2	-1.9

**11**	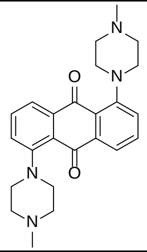	Fig. S11	-0.3	7.3	0.8	-4	3.8	-0.4
**12**	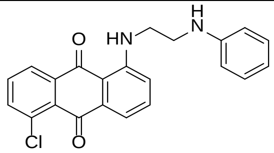	Fig. S11	-0.5	0	-1.4	-14.9	1.5	-2.2
**13**	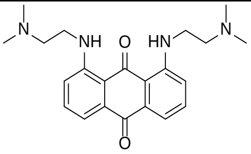	Fig. S11	8.6	12.3	3	18.5	17.2	6.1
**14**	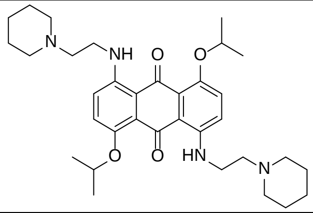	Fig. S12	-4.5	11	-3.8	4	-1	-1.5
**15**	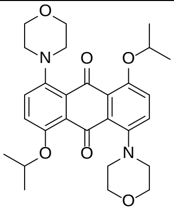	Fig. S12	-0.2	-1.3	0.4	-14	0.5	-2.2
**16**	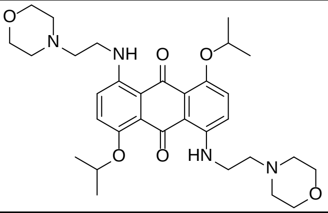	Fig. S12	-1.5	-0.9	-1.7	-0.1	1.3	-0.9
**17**	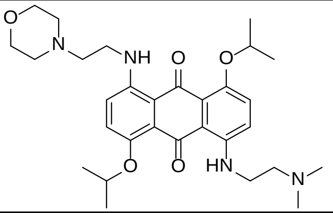	Fig. S12	-2	-0.8	-1.8	-0.4	1	-0.9
**18**	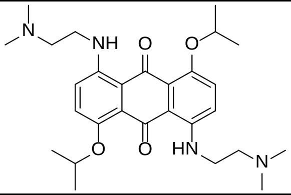	Fig. S12	0.6	-1.5	-1.4	-10	3.5	-2.3
**19**	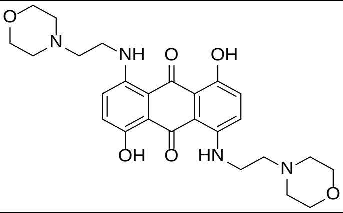	Fig. S12	1.2	-0.2	4.2	5.2	0.3	-0.8

**20**	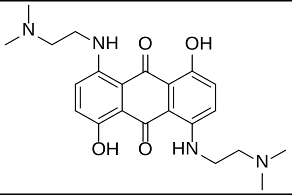	Fig. S12	5.4	19	5.7	20	16	6.8
**21**	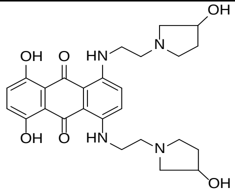	Fig. S13	29	38.5	30.2	30.9	18.6	6.2
**22**	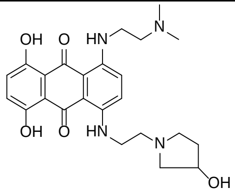	Fig. S13	35.2	40.5	28.2	35.2	21.3	8.5
**23**	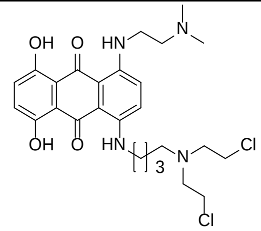	Previously described[3]	>49.4	>58	>44.2	>60	>49	>28.8
**24**	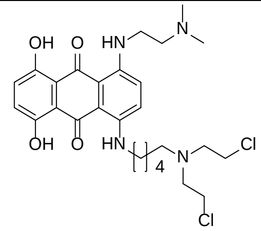	Previously described[3]	>49.2	>63.5	>44.2	>57.7	>46.8	>28.7
**25**	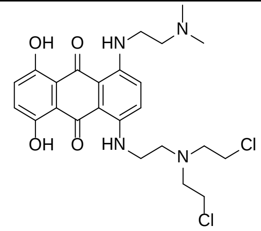	Previously described[3]	>49.3	>63.6	>44.3	>57.8	>46.9	>28.8

6. SYNTHETIC PROTOCOLS AND CHARACTERISATION OF MITOXANTRONE ANALOGUES

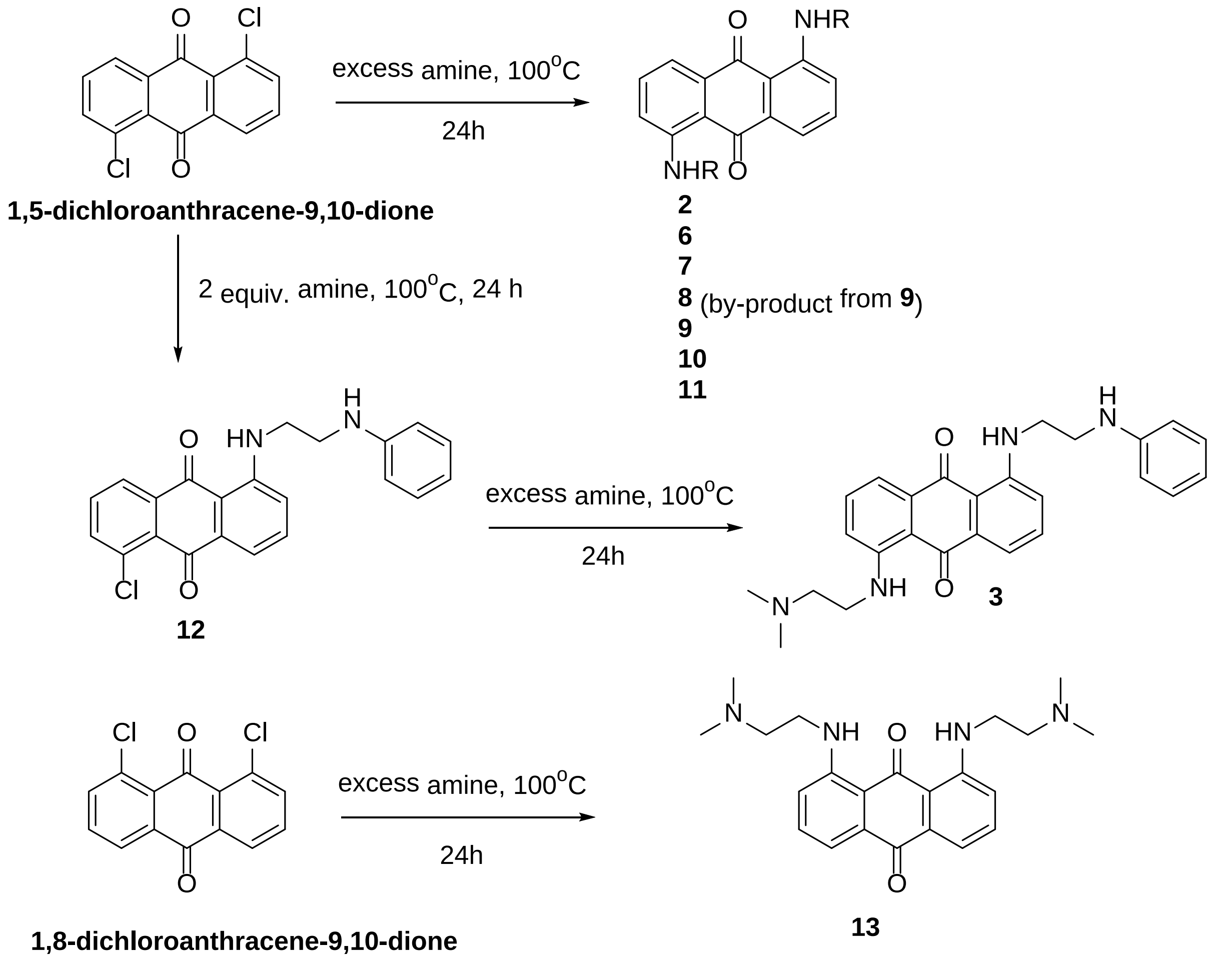

Figure S11: **General routes to 1-mono and 1,5-disubstituted aminoanthracene-9,10-diones**

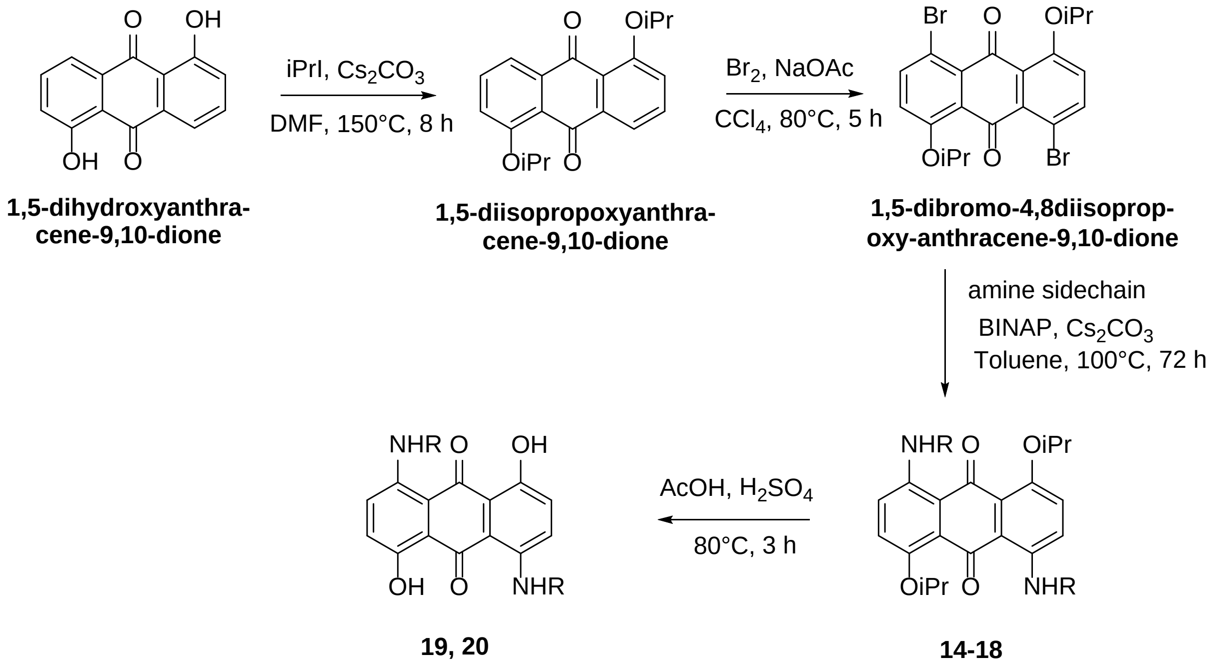

Figure S12: **General route to 1,5-dihydroxylated and isopropyl-protected aminoanthracene-9,10-diones**

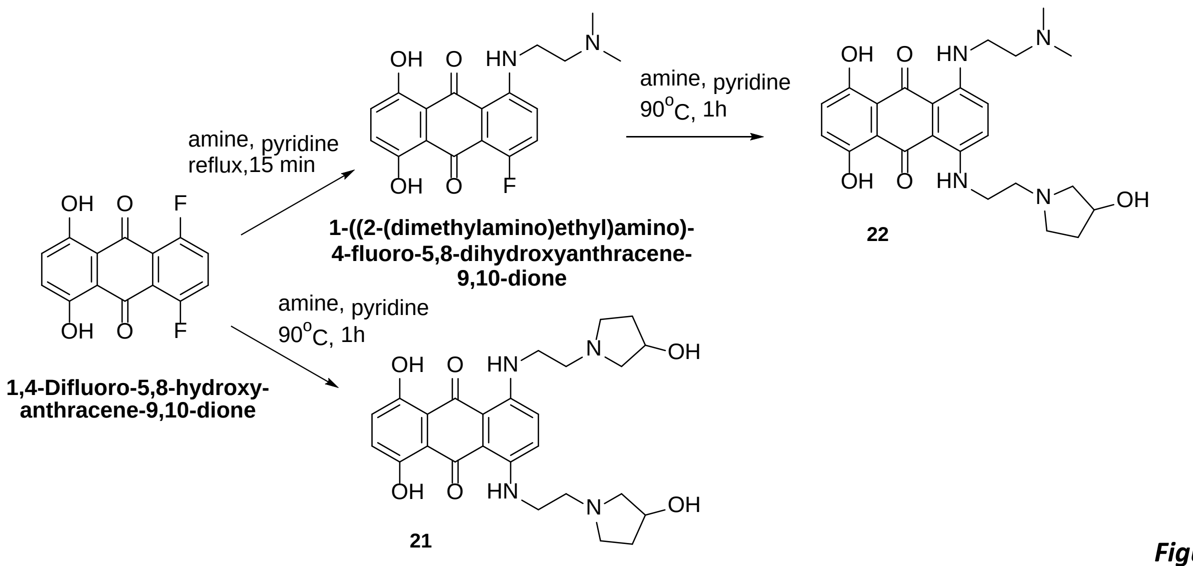

Figu re S13: **General route to 1,4-disubstituted aminoanthracene-9,10-diones**

1,5-bis((2-morpholinoethyl)amino)anthracene-9,10-dione (6)

The method follows that of **2** using 1,5-dichloroanthracene-9,10-dione (50 mg, 0.181 mmol) and 2-morpholinoethylamine (1 mL). The product **6** was afforded as a red solid (67.9 mg, 81%).

^{1}H NMR (400 MHz, $CDCl_3$): δ (ppm) 9.84 (s, 2H, Ar-*NH*), 7.60 (d, *J* = 7.2 Hz, 2H, *ArH*), 7.54 (d, *J* = 8.4 Hz, 2H, *ArH*), 6.98 (d, *J* = 8.4 Hz, 2H, *ArH*), 3.81 (m, 8H, $\mathit{CH_2}O\mathit{CH_2}$), 3.45 (dd, *J* = 6.4 Hz, 4H, NH$\mathit{CH_2}CH_2$) 2.76 (t, *J* = 6.4 Hz, 4H, NH$CH_2\mathit{CH_2}$), 2.58 (m, 8H, N$\mathit{CH_2}CH_2$O); ^{13}C NMR (101 MHz, $CDCl_3$): δ (ppm) 186.37 (2C, *C*=O), 151.37 (2C, C1 & C6), 136.35 & 113.27, 135.17, 116.36 & 114.91, 67.06, 56.96, 53.57, 39.93; m/z 465 ($[M+H]^+$, 100%); HRMS (*m/z*): $[M + H]^+$ calcd for $C_{26}H_{32}N_4O_4$, 465.2496; found, 465.2487.

1,5-bis((3-(dimethylamino)propyl)(methyl)amino)anthracene-9,10-dione (7)

The method follows that of **2** using 1,5-dichloroanthracene-9,10-dione (50 mg, 0.181 mmol) and N^1,N^1,N^2-trimethylethane-1,2-diamine (1 mL). The title compound **7** was afforded as a red powder (48.9 mg, 62%).

^{1}H NMR (400 MHz, $CDCl_3$): δ (ppm) 7.61 (d, *J* = 7.2 Hz, 2H, *ArH*), 7.43 (m, 2H, *ArH*), 7.19 (d, *J* = 8.2 Hz, 2H, *ArH*), 3.32 (t, 4H, *J* = 7.2 Hz, ArN$\mathit{CH_2}CH_2CH_2$), 2.82 (s, 6H, ArN$\mathit{CH_3}$), 2.24 (t, 4H, *J* = 7.2 Hz, ArN$CH_2CH_2\mathit{CH_2}$), 2.15 (12H, s, ArN$\mathit{CH_3}$), 1.80 (4H, dd, *J* = 7.2 Hz, ArN$CH_2\mathit{CH_2}CH_2$); ^{13}C NMR (101 MHz, $CDCl_3$): δ (ppm) 182.28, 151.67, 138.21, 120.20, 133.14, 121.35, 117.11, 56.91, 52.78, 45.47, 42.13, 25.44; m/z 437 ($[M+H]^+$, 64%); HRMS (*m/z*): $[M+H]^+$ calcd for $C_{26}H_{36}N_4O_2$, 437.2911; found, 437.2901.

1-chloro-5-((2-(1-methylpyrrolidin-3-yl)ethyl)amino)anthracene-9,10-dione (8)

The method follows that of **2** using 1,5-dichloroanthracene-9,10-dione (50 mg, 0.181 mmol) and 2-(1-methylpyrrolidin-3-yl)ethylamine (1 mL). The title compound, a by-product from the synthesis of **9**, was afforded as a red powder (12.7 mg, 19%).

^{1}H NMR (400 MHz, $CDCl_3$): δ (ppm) 9.56 (s, 1H, Ar-*NH*), 8.21 (d, *J* = 7.6 Hz, 1H, *ArH*), 7.55 (m, 4H, *ArH*), 6.97 (dd, *J* = 7.2 and 4.0 Hz, 1H, *ArH*), 3.33 (m, 2H, $NHCH_2CH_2$), 3.10 (t, *J* = 8.0 Hz, 1H), 2.34 (s, 3H, NCH_3), 1.5-2.27 (m, 8H); ^{13}C NMR (101 MHz, $CDCl_3$): δ (ppm) 183.38, 182.59, 151.29, 137.58, 135.76, 134.46, 129.36 & 112.27, 136.43, 135.17, 133.41, 126.23, 117.21 & 115.99, 64.40, 57.10, 40.52, 40.35, 32.67, 30.62, 22.03; m/z 369 ($[M+H]^+$, 100%); HRMS (*m/z*): $[M+H]^+$ calcd for $C_{21}H_{21}ClN_2O_2$, 369.1364; found, 369.1366.

1-((2-(1-methylpyrrolidin-2-yl)ethyl)amino)-5-((2-(1-methylpyrrolidin-3-yl)ethyl)amino)-anthracene-9,10-dione (9)

The method follows that of **2** using 1,5-dichloroanthracene-9,10-dione (50 mg, 0.181 mmol) and 2-(1-methylpyrrolidin-3-yl)ethylamine (1 mL). The title compound **9** was afforded as a red powder (50.7 mg, 61%).

^{1}H NMR (400 MHz, $CDCl_3$): δ (ppm) 9.64 (t, *J* = 4.8 Hz, 1H, Ar-*NH*), 7.46 (m, 4H, *ArH*), 6.90 (d, *J* = 8.0 Hz, 2H, *ArH*), 3.33 (m, 4H, $NHCH_2CH_2$), 3.10 (t, *J* = 8.6 Hz, 2H), 2.31 (s, 6H, NCH_3), 2.25-1.5 (m, 16H). ^{13}C NMR (101 MHz, $CDCl_3$): δ (ppm) 185.45, 151.34, 136.29, 112.95, 135.24, 116.34, 114.78, 64.31, 57.16, 40.54, 40.35, 32.91, 30.68, 22.00; m/z 361 ($[M+H]^+$, 30%); HRMS (*m/z*): $[M+H]^+$ calcd for $C_{28}H_{36}N_4O_2$, 461.2911; found, 461.2908.

1,5-dimorpholinoanthracene-9,10-dione (10)

The method follows that of **2** using 1,5-dichloroanthracene-9,10-dione (50 mg, 0.181 mmol) and morpholine (1 mL). The product **10** was afforded as a red powder (47.8 mg, 70 %).

^{1}H NMR (400 MHz, $CDCl_3$): δ (ppm) 7.84 (d, *J* = 7.6 Hz, 2H, *ArH*), 7.57 (t, *J* = 8.0 Hz, 2H, *ArH*), 7.22 (d, *J* = 8.4 Hz, 2H, *ArH*), 3.94 (m, 8H, NCH_2CH_2O), 3.12 (m, 8H, NCH_2CH_2O); ^{13}C NMR (101 MHz, $CDCl_3$): δ (ppm) 181.51, 151.50, 137.63, 121.14, 133.47, 121.95, 119.95, 66.01, 51.81; m/z 379 ([M+H]+, 100%); HRMS (*m/z*): $[M + H]^+$ calcd for $C_{22}H_{22}N_2O_4$, 379.1652; found, 379.1653.

1,5-bis(4-methylpiperazin-1-yl)anthracene-9,10-dione (11)

The method follows that of **2** using 1,5-dichloroanthracene-9,10-dione (50 mg, 0.181 mmol) and *N*-methylpiperazine (1 mL). The title compound **11** was afforded as a red powder (48.9 mg, 67%).

^{1}H NMR (400 MHz, $CDCl_3$): δ (ppm) 7.82 (d, *J* = 8.8 Hz, 2H, *ArH*), 7.53 (dd, *J* = 8.0 Hz, 2H, *ArH*), 7.23 (d, *J* = 8.4 Hz, 2H, *ArH*), 3.16 (m, 8H, ArN$CH_2$$CH_2$), 2.68 (m, 8H, ArN$CH_2$$CH_2$), 2.35 (s, 6H, N$CH_3$); ^{13}C NMR (101 MHz, $CDCl_3$): δ (ppm) 182.58, 152.52, 138.63, 122.05, 134.32, 123.12, 120.60, 55.12, 52.35, 46.12; m/z 405 ($[M+H]^+$, 20%); HRMS (*m/z*): $[M+H]^+$ calcd for $C_{24}H_{28}N_4O_2$, 405.2285; found, 405.2277.

1-chloro-5-((2-(phenylamino)ethyl)amino)anthracene-9,10-dione (12)

The method follows that of **2** using 1,5-dichloroanthracene-9,10-dione (50 mg, 0.181 mmol), *N^1*-phenylethane-1,2-diamine (49.6 µL, 0.379 mmol) and pyridine (1 mL). The title compound **12** was afforded as a red powder (35.4 mg, 52%).

^{1}H NMR (400 MHz, $CDCl_3$): δ (ppm) 9.68 (t, *J* = 4.8 Hz, 1H, Ar*NH*), 8.21 (d, *J* = 7.6 Hz, 1H, *ArH*), 7.65 (d, *J* = 7.2 Hz, 1H, *ArH*), 7.53 (t, *J* = 7.6 Hz, 1H, *ArH*), 7.49 (m, 2H, *ArH*), 7.14 (t, *J* = 7.6 Hz, 2H, *ArH*), 6.99 (d, *J* = 7.2 Hz, 1H, *ArH*), 6.68 (t, *J* = 7.6 Hz, 1H, *ArH*), 6.62 (d, *J* = 8.0 Hz, 2H, *ArH*), 3.88 (br, s, 1H, CH_2*NH*Ph), 3.56 (q, *J* = 6.0 Hz, 2H, NH$CH_2$$CH_2$NHPh), 3.47 (t, *J* = 6.0 Hz, 2H, NH$CH_2$$CH_2$NHPh); ^{13}C NMR (101 MHz, $CDCl_3$): δ (ppm) 183.85, 182.51, 151.41, 147.35, 137.29, 136.56, 135.85, 134.36, 133.49, 129.42, 126.30, 120.84, 119.47, 118.05, 117.19, 116.37, 113.06, 113.05, 43.11, 42.20; m/z 377 ($[M+H]^+$, 100%); HRMS (*m/z*): $[M+H]^+$ calcd for $C_{22}H_{17}ClN_2O_2$, 377.1051; found, 377.1054.

1,8-bis((2-(dimethylamino)ethyl)amino)anthracene-9,10-dione (13)

The method follows that of **2** using 1,8-dichloroanthracene-9,10-dione (50 mg, 0.181 mmol) and *N*,*N*-dimethylethane-1,2-diamine (1 mL). The title compound **13** was afforded as a red powder (35 mg, 51%).

^{1}H NMR (400 MHz, $CDCl_3$): δ (ppm) 9.65 (s, 2H, Ar-*NH*), 7.60 (d, *J* = 7.2 Hz, 2H, *ArH*), 7.44 (m, 4H, *ArH*), 6.94 (d, *J* = 8.4 Hz, 2H, *ArH*), 3.34 (q, *J* = 6.4 Hz, 4H, NH$CH_2$$CH_2$) 2.61 (t, *J* = 6.4 Hz, 4H, NH$CH_2$$CH_2$), 2.29 (s, 12H, N-$CH_3$); ^{13}C NMR (101 MHz, $CDCl_3$): δ (ppm) 184.92, 150.98, 134.38, 134.11,

117.60, 114.86, 114.63, 58.10, 45.68, 41.19; m/z 381 ($[M+H]^+$, 100%); HRMS (*m/z*): $[M+H]^+$ calcd for $C_{22}H_{28}N_4O_2$, 381.2285; found, 381.2287.

1,5-diisopropoxyanthracene-9,10-dione

To a stirred solution of 1,5-dihydroxyanthracene-9,10-dione (2.0 g, 8.326 mmol, 1 eq.) in anhydrous DMF (17 mL) was added cesium carbonate (40.69 g, 124.88 mmol, 15 eq.). The resulting mixture was stirred and heated at 150°C under N_2. 2-iodopropane (25 mL, 249.77 mmol, 30 eq.) was added in portions of 3 mL over 8 hours. The purple colored reaction mixture was left to stir overnight at 150°C. After cooling to room temperature, the mixture was filtered and washed with CH_2Cl_2 through a short pad of silica to remove the cesium carbonate. The resulting orange/brown solution was concentrated *in vacuo* to afford a brown residue, which was purified by flash column chromatography using EtOAc:PE (1:9) to yield the title compound (2.36 g, 87%) as an orange/brown solid.

^{1}H NMR (400 MHz, $CDCl_3$): δ (ppm) 7.87 (2H, dd, *J* = 7.0, 1.5 Hz, *ArH*), 7.64 (2H, t, *J* = 7.9 Hz, *ArH*), 7.25 (2H, d, *J* = 8.1 Hz, *ArH*), 4.71 (2H, m, C*H*-$(CH_3)_2$), 1.48 (12H, d, *J* = 6.0 Hz, CH-$(C\mathit{H_3})_2$); ^{13}C NMR (100 MHz, $CDCl_3$): δ (ppm) 182.5.1, 158.4, 137.8, 134.6, 122.1, 120.0, 119.7, 72.3, 22.1; m/z 325 ($[M+H]^+$, 100%).

1,5-dibromo-4,8-diisopropoxyanthracene-9,10-dione

To a stirred solution of **1,5-diisopropoxylanthracene-9,10-dione** (1.0 g, 3.08 mmol, 1 eq.) in CCl_4 (25 mL) was added sodium acetate (1.64 g, 20.04 mmol, 6.5 eq.). The resulting solution was heated at 80°C and bromine (2.53 mL, 49.34 mmol, 16 eq.) in CCl_4 (6.5 mL) was added dropwise within 30 minutes. The resulting solution was heated at 80°C for 5 hours before it was concentrated *in vacuo* to give an orange residue. The resulting solid was purified by flash column chromatography using EtOAc:PE (1:9→2:8) to yield the title compound (0.99 g, 67%) as an orange solid.

^{1}H NMR (400 MHz, $CDCl_3$): δ (ppm) 7.71 (2H, d, *J* = 9.0, *ArH*), 7.02 (2H, d, *J* = 9.0 Hz, *ArH*), 4.58 (2H, m, C*H*-$(CH_3)_2$), 1.41 (12H, d, *J* = 6.1 Hz, CH-(C*H*$_3$)$_2$); ^{13}C NMR (100 MHz, $CDCl_3$): δ (ppm) 182.2, 156.2, 139.5, 135.9, 126.8, 120.9, 110.1, 73.5, 21.9; m/z 481/483/485 ($[M+H]^+$, 50%).

1,5-diisopropoxy-4,8-bis((2-(piperidin-1-yl)ethyl)amino)anthracene-9,10-dione (14)

Toluene (4 mL) was added to palladium acetate (4.4 mg, 0.019 mmol) and BINAP ((±)-2,2′-Bis(diphenylphosphino)-1,1′-binaphthalene) (15.2 mg, 0.025 mmol) under argon. The resulting solution was degassed by bubbling argon through the solution for 30 minutes. 1,5-dibromo-4,8-diisopropoxyanthracene-9,10-dione (49 mg, 0.099 mmol), cesium carbonate (128.6 mg, 0.395 mmol) and 2-(piperidin-1-yl)ethylmine (1 mL) were then added to the solution, which was stirred and heated at 100°C for 72 hours under argon. The reaction mixture was then cooled to room temperature before it was filtered through a short pad of celite to remove the palladium and the cesium carbonate. The remaining solution was concentrated *in vacuo* and the resulting residue was purified by flash column chromatography using CH_2Cl_2:CH_3OH (95:5→90:10) to yield the title compound **14** (41.1 mg, 72%) was afforded as a dark purple solid.

^{1}H NMR (400 MHz, $CDCl_3$): δ (ppm) 9.50 (t, *J* = 4.72 Hz, 2 H, Ar-*NH*), 7.11 (d, *J* = 9.2 Hz, 2H, *ArH*), 6.83 (d, *J* = 9.2 Hz, 2H, *ArH*), 4.23 (hept, *J* = 6.0 Hz, 2H, isopropyl-*CH*), 3.31 (m, 4 H, ArNH*CH*$_2$CH$_2$), 2.60 (t, *J* = 6.8 Hz, 4H, ArNHCH$_2$*CH*$_2$), 2.42 (m, 8H, N*CH*$_2$CH$_2$CH$_2$), 1.57 (m, 8H, NCH$_2$*CH*$_2$CH$_2$), 1.42 (m, 4H, NCH$_2$CH$_2$*CH*$_2$), 1.29 (d, *J* = 6.0 Hz, 12H, isopropyl-*CH*$_3$); ^{13}C NMR (101 MHz, $CDCl_3$): δ (ppm) 186.11, 147.52, 146.60, 130.58, 117.16, 126.31, 114.52, 75.26, 57.64, 54.68, 40.51, 26.03, 24.42, 22.41; m/z 577 ($[M+H]^+$, 40%); HRMS (*m/z*): $[M+H]^+$ calcd for $C_{34}H_{48}N_4O_4$, 577.3748; found, 577.3738.

1,5-diisopropoxy-4,8-dimorpholinoanthracene-9,10-dione (15)

The method follows that of **14** using palladium acetate (6.2 mg, 0.027 mmol), BINAP ((±)-2,2′-Bis(diphenylphosphino)-1,1′-binaphthalene) (21.3 mg, 0.035 mmol), 1,5-dibromo-4,8-diisopropoxyanthracene-9,10-dione (69 mg, 0.138 mmol), cesium carbonate (180 mg, 0.553 mmol)

and morpholinoethylamine (1 mL). The title compound **15** (46.5 mg, 66%) was afforded as a dark purple solid.

^{1}H NMR (400 MHz, $CDCl_3$): δ (ppm) 7.03 (m, 4H, *ArH*), 4.42 (hept, *J* = 6.0 Hz, 2H, isopropyl-*CH*), 3.83 (m, 8H, $NCH_2\mathit{CH_2}O$), 3.00 (m, 8 H, $N\mathit{CH_2}CH_2O$), 1.29 (d, *J* = 6.0 Hz, 12H, isopropyl-$\mathit{CH_3}$); ^{13}C NMR (101 MHz, $CDCl_3$): δ (ppm) 185.73, 150.58, 144.64, 129.11, 128.53, 123.72, 122.26, 73.30, 67.11, 53.20, 22.16; m/z 413 ([M+H]$^+$, 100%); HRMS (*m/z*): [M+H]$^+$ calcd for $C_{28}H_{34}N_2O_6$, 495.2490; found, 495.2481.

1,5-diisopropoxy-4,8-bis((2-morpholinoethyl)amino)anthracene-9,10-dione (16)

The method follows that of **14** using palladium acetate (9.3 mg, 0.041 mmol), BINAP ((±)-2,2′-Bis(diphenylphosphino)-1,1′-binaphthalene) (32 mg, 0.052 mmol), 1,5-dibromo-4,8-diisopropoxyanthracene-9,10-dione (103 mg, 0.207 mmol), cesium carbonate (270 mg, 0.829 mmol) and morpholino (1 mL). The title compound **16** (61 mg, 61%) was afforded as a dark purple solid.

^{1}H NMR (400 MHz, $CDCl_3$): δ (ppm) 9.65 (t, *J* = 4.4 Hz, 2H, Ar-*NH*), 7.21 (d, *J* = 9.2 Hz, 2H, *ArH*), 6.91 (d, *J* = 9.2 Hz, 2H, *ArH*), 4.33 (hept, *J* = 6.0 Hz, 2H, isopropyl-*CH*), 3.81 (m, 8H, $\mathit{CH_2}O\mathit{CH_2}$), 3.40 (q, *J* = 6.8 Hz, 4H, $NH\mathit{CH_2}CH_2$) 2.74 (t, *J* = 6.8 Hz, 4H, $NHCH_2\mathit{CH_2}$), 2.58 (m, 8H, $\mathit{CH_2}CH_2OCH_2\mathit{CH_2}$), 1.38 (d, *J* = 6.0 Hz, 12H, isopropyl-$\mathit{CH_3}$). ^{13}C NMR (101 MHz, $CDCl_3$): δ (ppm) 185.48, 147.00, 145.78, 129.81, 116.54, 125.62, 114.01, 74.55, 66.42, 56.43, 52.91, 39.27, 21.83; m/z 581 ([M+H]$^+$, 52%); HRMS (*m/z*): [M+H]$^+$ calcd for $C_{32}H_{44}N_4O_6$, 581.3334; found, 581.3328.

1-((2-(dimethylamino)ethyl)amino)-4,8-diisopropoxy-5-((2-morpholinoethyl)amino)anthracene-9,10-dione (17)

The method is described under **16** and is a by-product, which was afforded as a dark purple solid (15.6 mg, 14 %).

^{1}H NMR (400 MHz, $CDCl_3$): δ (ppm) 9.56 (t, *J* = 4.8 Hz, 1H, Ar*NH*), 9.49 (t, *J* = 4.8 Hz, 1H, Ar*NH*), 7.13 (d, *J* = 9.6 Hz, 1H, *ArH*), 7.12 (d, *J* = 9.6 Hz, 1H, *ArH*), 6.84 (t, *J* = 9.6 Hz, 1H, *ArH*), 6.82 (d, *J* = 9.6 Hz, 1H, *ArH*), 4.33 (m, 2H, isopropyl-*CH*), 3.72 (m, 8H, $\mathit{CH_2}O\mathit{CH_2}$), 3.31 (m, 4H, $NH\mathit{CH_2}CH_2$), 2.65 (t, *J* = 6.8

Hz, 2H, $NHCH_2CH_2$), 2.59 (t, *J* = 6.8 Hz, 2H, $NHCH_2CH_2$), 2.49 (m, 4H, $CH_2CH_2OCH_2CH_2$), 2.28 (s, 6H, NCH_3), 1.30 (d, *J* = 6.0 Hz, 6H, isopropyl-CH_3), 1.28 (d, *J* = 6.0 Hz, 6H, isopropyl-CH_3). ^{13}C NMR (101 MHz, $CDCl_3$): δ (ppm) 186.20, 186.09, 147.00, 146.53, 146.45, 130.61, 130.44, 117.18, 117.11, 126.36, 126.17, 114.62, 114.58, 75.49, 75.14, 67.06, 58.10, 57.06, 53.54, 45.56, 41.11, 39.90, 22.45, 22.35; m/z 539 ($[M+H]^+$, 30%); HRMS (*m/z*): $[M+H]^+$ calcd for $C_{30}H_{42}N_4O_5$, 539.3228; found, 539.3218.

1,5-bis((2-(dimethylamino)ethyl)amino)-4,8-diisopropoxyanthracene-9,10-dione (18)

The method follows that of **14** using palladium acetate (3.72 mg, 0.016 mmol), BINAP ((±)-2,2′-Bis(diphenylphosphino)-1,1′-binaphthalene) (12.8 mg, 0.021 mmol), 1,5-dibromo-4,8-diisopropoxyanthracene-9,10-dione (41.4 mg, 0.083 mmol), cesium carbonate (108 mg, 0.332 mmol) and *N*,*N*-dimethylethane-1,2-diamine (1 mL). The title compound **19** (32.6 mg, 79%) was afforded as a dark purple solid.

^{1}H NMR (400 MHz, $CDCl_3$): δ (ppm) 9.48 (t, *J* = 4.8 Hz, 2 H, *Ar-NH*), 7.12 (d, *J* = 9.2Hz, 2H, *ArH*), 6.83 (d, *J* = 9.2 Hz, 2H, *ArH*), 4.21 (hept, *J* = 6.0 Hz, 2H, isopropyl-*CH*), 3.31 (q, *J* = 6.8 Hz, 4 H, $NHCH_2CH_2$), 2.58 (t, *J* = 6.8 Hz, 4H, $NHCH_2CH_2$), 2.28 (s, 12H, NCH_3), 1.28 (d, *J* = 6.0 Hz, 12H, isopropyl-CH_3); ^{13}C NMR (101 MHz, $CDCl_3$): δ (ppm) 186.19, 147.69, 146.56, 126.27, 114.58, 130.57, 117.13, 75.44, 58.11, 45.57, 41.12, 22.35; m/z 497 ($[M+H]^+$, 40%); HRMS (*m/z*): $[M+H]^+$ calcd for $C_{28}H_{40}N_4O_4$, 497.3122; found, 497.3119.

1,5-dihydroxy-4,8-bis((2-morpholinoethyl)amino)anthracene-9,10-dione (19):

To a stirred solution of **14** (75 mg, 0.129 mmol) in acetic acid (25 mL) was dropwise added sulphuric acid (0.5 mL) over a period of 10 min. The resulting mixture was stirred at 80°C for 3 hours before it was allowed to cool to room temperature and concentrated *in vacuo*. The resulting residue was purified by flash column chromatography on silica gel using CH_2Cl_2:CH_3OH (95:5→90:10 + 0.1% conc. NH_3 ammonia) to yield the title compound **19** (48 mg, 75%) as a dark blue solid.

^{1}H NMR (400 MHz, $CDCl_3$): δ (ppm) 14.01 (s, 2H, Ar-*OH*), 9.88 (t, *J* = 4.6 Hz, 2H, Ar*NH*), 7.15 (d, *J* = 9.6 Hz, 2H, *ArH*), 6.99 (d, *J* = 9.6 Hz, 2H, *ArH*), 3.71 (m, 8H, CH_2OCH_2), 3.39 (q, *J* = 6.4 Hz, 4H, $NHCH_2CH_2$)

2.67 (t, J = 6.4 Hz, 4H, NHCH$_2$*CH$_2$*), 2.49 (m, 8H, *CH$_2$*CH$_2$OCH$_2$*CH$_2$*). ^{13}C NMR (101 MHz, $CDCl_3$): δ (ppm) 186.69, 155.19, 146.87, 128.76, 115.21, 120.99, 109.66, 67.09, 56.83, 53.46, 39.94; m/z 497 ([M+H]$^+$, 100%); HRMS (*m/z*): [M+H]$^+$ calcd for $C_{26}H_{32}N_4O_6$, 497.2395; found, 497.2388.

1,5-bis((2-(dimethylamino)ethyl)amino)-4,8-dihydroxyanthracene-9,10-dione (20)

The method follows that of **14** using **18** (25 mg, 0.050 mmol), acetic acid (20 mL) and sulphuric acid (0.5 mL). The product **20** was afforded as a dark blue powder (11.3 mg, 55 %).

^{1}H NMR (400 MHz, $CDCl_3$): δ (ppm) 13.95 (s, 2H, Ar*OH*), 9.74 (t, J = 4.4 Hz, 2H, Ar*NH*), 7.14 (d, J = 9.2 Hz, 2H, *ArH*), 6.98 (d, J = 9.2 Hz, 2H, *ArH*), 3.37 (q, J = 6.4 Hz, 4H, NH*CH$_2$*CH$_2$), 2.59 (t, J = 6.4 Hz, 4H, NHCH$_2$*CH$_2$*), 2.27 (s, 12H, N*CH$_3$*); ^{13}C NMR (101 MHz, $CDCl_3$): δ (ppm) 186.64 (2C, *C*=O), 155.11 (2C, C4 & C9), 147.00 (2C, C1 & C6), 128.76 & 120.82 (4C, C2, C3, C7 & C8), 115.18 & 109.57 (4C, C11, C12, C13 & C14), 58.21 (2C, NH*CH$_2$*CH$_2$), 45.64 (4C, N*CH$_3$*), 41.16 (2C, NHCH$_2$*CH$_2$*); m/z 413 ([M+H]$^+$, 100%); HRMS (*m/z*): [M+H]$^+$ calcd for $C_{22}H_{28}N_4O_4$, 413.2183; found, 413.2183.

1,4-Bis-([2-(3-hydroxypyrrolidine)ethyl]amino)-5,8-dihydroxy-anthracene-9,10-dione (21)

1,4-Difluoro-5,8-hydroxyanthracene-9,10-dione (75 mg, 0.272 mmol) and 1-(2-aminoethyl)-pyrrolidin-3-ol (1 g, 7.7 mmol) were stirred in pyridine (2 mL) at 90 °C for 1 h. The reaction mixture was added to ice-cold brine and set aside at 4°C overnight. The precipitated solid was isolated by filtration and lyophilised. The desired product was purified by flash chromatography, initially eluting with CH_2Cl_2/CH_3OH (95:5) to remove non-polar impurities, followed by a gradual increase of CH_2Cl_2 to CH_2Cl_2/CH_3OH (85:15). The chromatographed product was then crystallised from $CHCl_3$ affording the title compound **21** was afforded as a dark blue powder (59.2 mg, 44%) as a dark blue powder (0.11 g, 34%).

^{1}H NMR (270 MHz, DMSO/$CDCl_3$(1:1): δ (ppm) 13.55 (s, 2H, ArO*H*), 10.55 (t, J = 4.4 Hz, 2H, ArN*H*), 7.3 (m, 2H, Ar*H*), 7.05 (s, 2H, Ar*H*), 4.10 (m, 2H, CH$_2$C*H*OH), 3.60 (q, J = 7.0 Hz 4H, HNC*H$_2$*CH$_2$N), 2.80 (t, q, J = 7.0 Hz 4H, HNCH$_2$C*H$_2$*N), 2.75 (m, 10H, ring-*H* and O*H*), 2.05 (m, 2H, ring-*H*), 1.60 (m, 2H, ring-*H*); ^{13}C NMR (62.9 MHz, DMSO/$CDCl_3$(1:1): δ (ppm) 183.21, 154.38, 146.43, 124.90, 123.79,

115.04, 107.01, 69.26, 62.33, 54.42, 52.18, 41.22, 34.73, 28.01; m/z 497 ($[M+H]^+$, 100%); Anal. Calcd for $C_{26}H_{32}N_4O_6$: C, 62.88; H, 6.51; N, 11.28. Found: C, 62.50; H, 6.54; N, 11.00.

1-([(2-Dimethylamino)ethyl]amino)-4-([2-(3-hydroxypyrrolidine)ethyl]amino)-5,8-dihydroxy-anthracene-9,10-dione (22)

The method follows that of **21** using 1-(2-Dimethylamino)ethylamino-4-fluoro-5,8-dihydroxy-anthracene-9,10-dione (45 mg, 0.13 mmol), 1-(2-aminoethyl)-pyrrolidin-3-ol (880 mg, 6.77 mmol), pyridine (1 mL). The product **22** was afforded as a dark blue powder (24 mg, 41 %)

^{1}H NMR (270 MHz, DMSO/$CDCl_3$(1:1): δ (ppm) 13.5 (s, 2H, ArO*H*) 10.5 (t, *J* = 4.4 Hz, 2H, ArN*H*), 7.4 (m, 2H, Ar*H*), 7.1 (s, 2H, Ar*H*), 4.25 (m, 1H, CH_2C*H*OH), 3.53 (q, *J* = 6.8 Hz, 4H, HNC$H_2$$CH_2$N), 2.75 (m, 2H, ring-*H* and O*H*), 2.55 (t, *J* = 6.8 Hz, 4H, HNCH2CH_2N), 2.38 (m, 1H, ring-*H*), 2.3 (s, 6H, NCH_3), 2.00 (m, 2H, ring-*H*), 1.62 (m, 1H, ring-*H*); ^{13}C NMR (62.9 MHz, DMSO/$CDCl_3$(1:1): δ (ppm) 183.16, 154.38, 146.90, 124.90, 123.79, 114.78, 107.18, 69.26, 62.33, 54.42, 52.18, 41.22, 34.19; m/z 455 ($[M+H]^+$, 100%).

7. HPLC TRACES

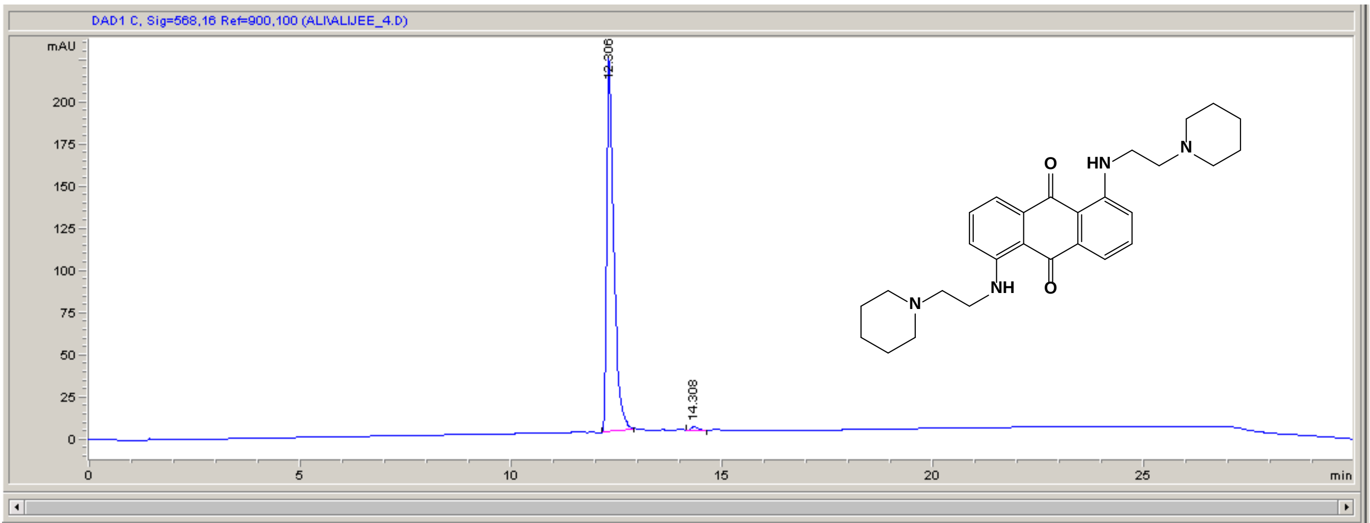

File Information

LC-File	ALIJEE_4.D
File Path	C:\CHEM32\1\DATA\ALI\
Date	16-Jan-16, 09:26:19
Sample	Jee2
Sample Info	
Barcode	
Operator	ali
Method	ALIAQ.M
Analysis Time	30 min
Sampling Rate	0.0067 min (0.402 sec), 4501 datapoints

#	Time	Area	Height	Width	Area%	Symmetry
1	12.306	2695.2	221.9	0.1797	98.763	0.481
2	14.308	33.7	2.5	0.1976	1.237	0.595

Compound 2

Compound 3

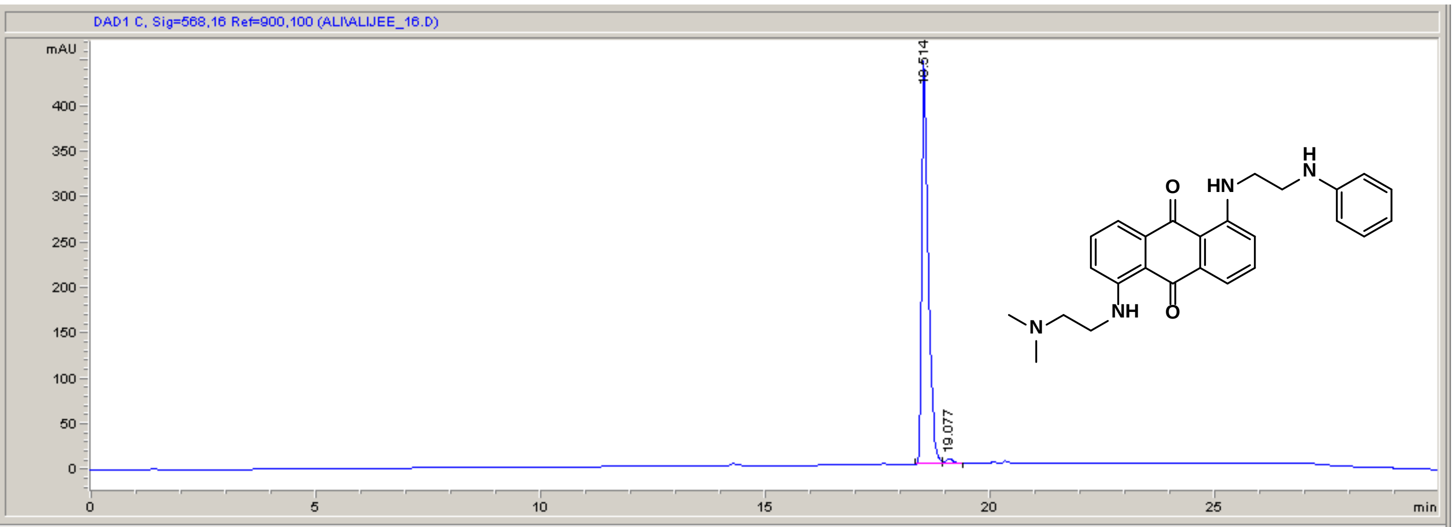

File Information

LC-File	ALIJEE_16.D
File Path	C:\CHEM32\1\DATA\ALI\
Date	17-Jan-16, 09:33:59
Sample	Jee25
Sample Info	
Barcode	
Operator	ali
Method	ALIAQ.M
Analysis Time	30 min
Sampling Rate	0.0067 min (0.402 sec), 4501 datapoints

#	Time	Area	Height	Width	Area%	Symmetry
1	18.514	4656.8	444.1	0.1603	98.559	0.52
2	19.077	68.1	6.1	0.156	1.441	0.675

Compound 6

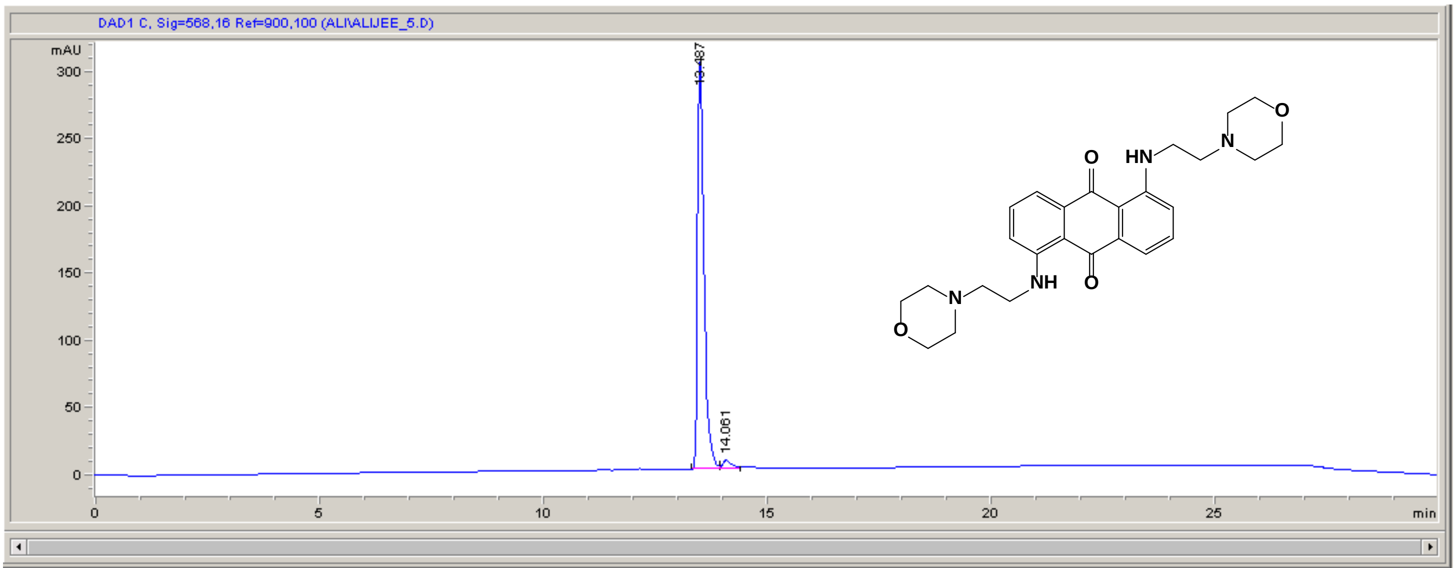

File Information

LC-File	ALIJEE_5.D
File Path	C:\CHEM32\1\DATA\ALI\
Date	16-Jan-16, 10:04:30
Sample	Jee3
Sample Info	
Barcode	
Operator	ali
Method	ALIAQ.M
Analysis Time	30 min
Sampling Rate	0.0067 min (0.402 sec), 4501 datapoints

#	Time	Area	Height	Width	Area%	Symmetry
1	13.487	3010.7	302.8	0.148	97.115	0.621
2	14.061	89.4	6.9	0.1843	2.885	0.538

Compound 7

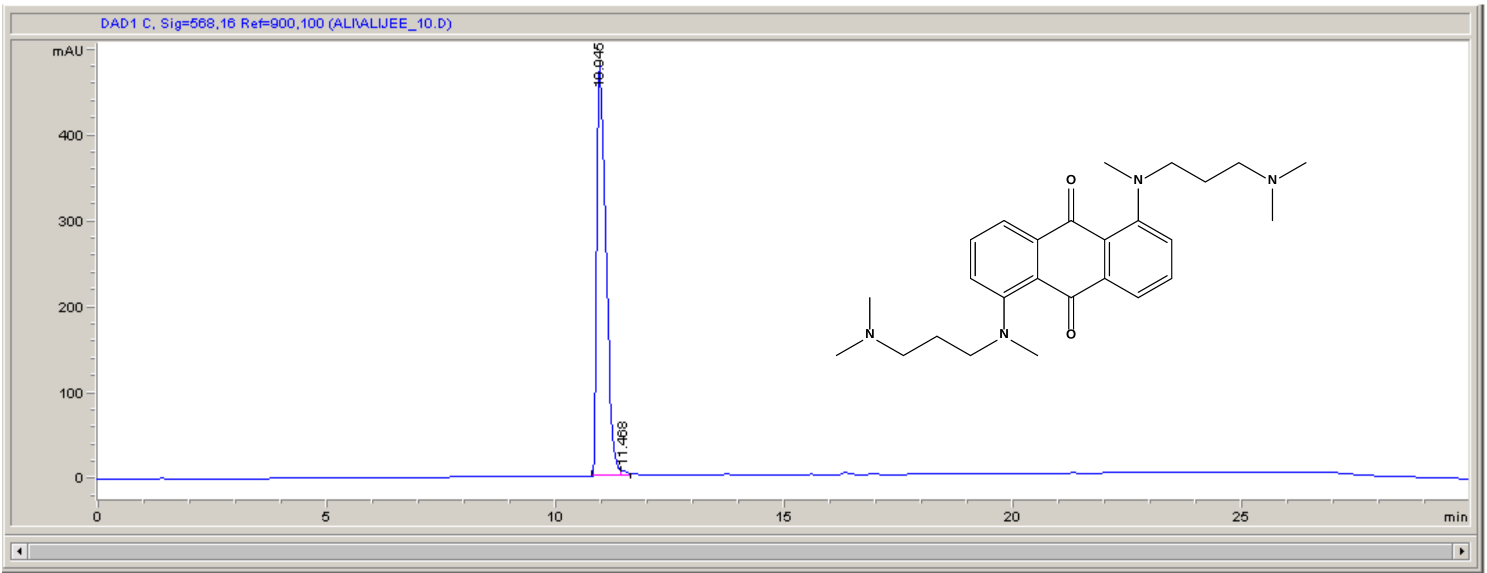

File Information

LC-File	ALIJEE_10.D
File Path	C:\CHEM32\1\DATA\ALI\
Date	16-Jan-16, 13:23:48
Sample	Jee7
Sample Info	
Barcode	
Operator	ali
Method	ALIAQ.M
Analysis Time	30 min
Sampling Rate	0.0067 min (0.402 sec), 4501 datapoints

#	Time	Area	Height	Width	Area%	Symmetry
1	10.945	6870.1	480.6	0.2308	99.273	0.418
2	11.468	50.3	5.3	0.1358	0.727	0.324

Compound 8

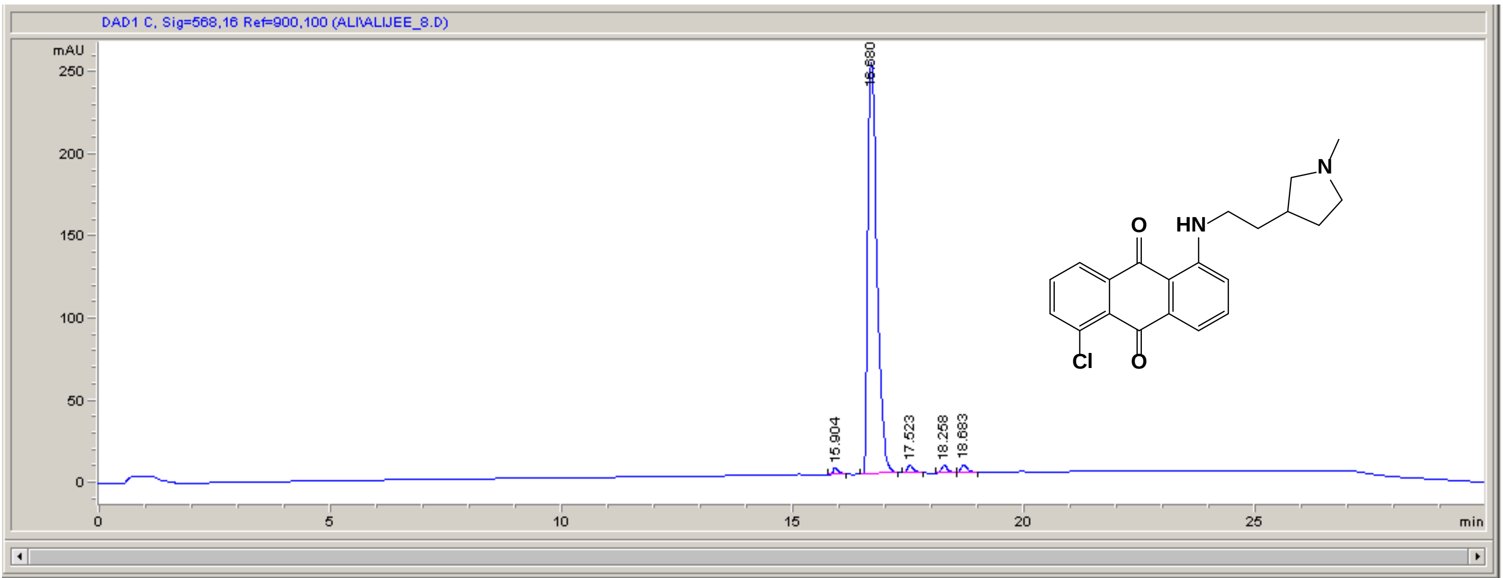

File Information

LC-File	ALIJEE_8.D
File Path	C:\CHEM32\1\DATA\ALI\
Date	16-Jan-16, 11:57:51
Sample	Jee5m
Sample Info	
Barcode	
Operator	ali
Method	ALIAQ.M
Analysis Time	29.993 min
Sampling Rate	0.0067 min (0.402 sec), 4500 datapoints

#	Time	Area	Height	Width	Area%	Symmetry
1	15.904	35.3	3.8	0.1388	0.998	0.602
2	16.68	3363.3	250.4	0.2127	94.979	0.471
3	17.523	52.1	4.7	0.1595	1.473	0.579
4	18.258	43.7	4.5	0.1428	1.233	0.608
5	18.683	46.6	4.7	0.1446	1.317	0.612

Compound 9

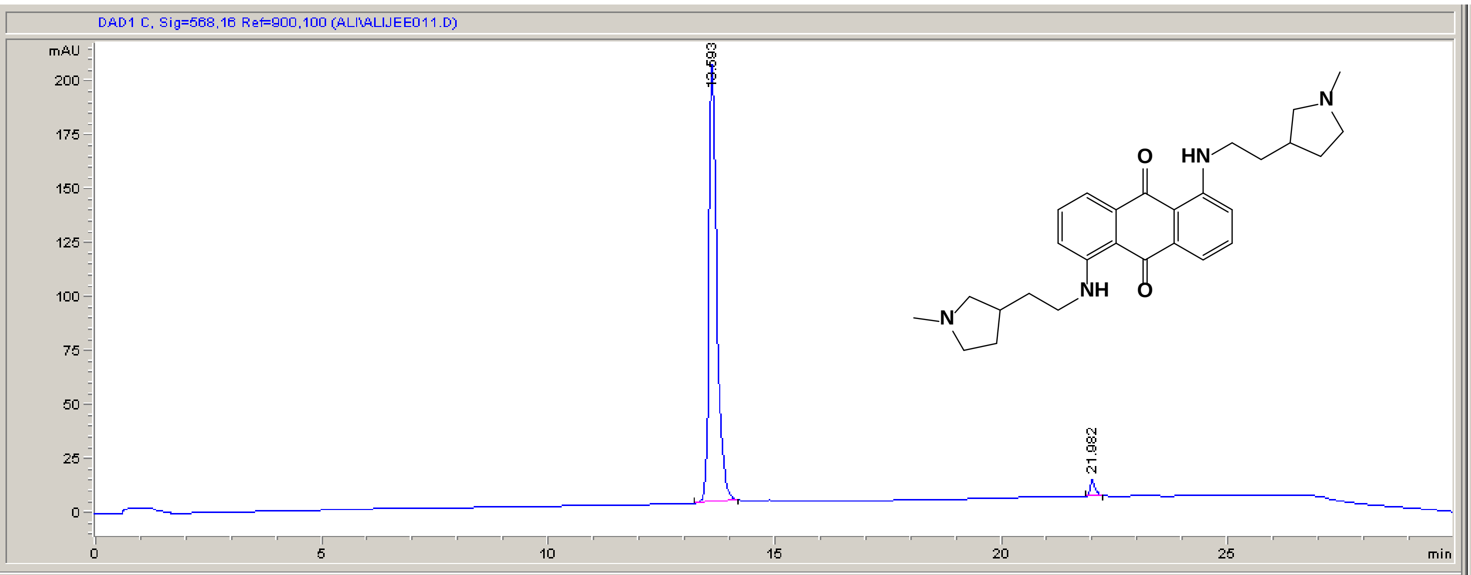

File Information

LC-File	ALIJEE011.D
File Path	C:\CHEM32\1\DATA\ALI\
Date	03-Feb-16, 08:12:30
Sample	JEE5DI Pd
Sample Info	
Barcode	
Operator	ALI
Method	ALIAQ.M
Analysis Time	30 min
Sampling Rate	0.0067 min (0.402 sec), 4501 datapoints

#	Time	Area	Height	Width	Area%	Symmetry
1	13.593	2454	203.1	0.177	97.422	0.582
2	21.982	64.9	8.2	0.1165	2.578	0.594

Compound 10

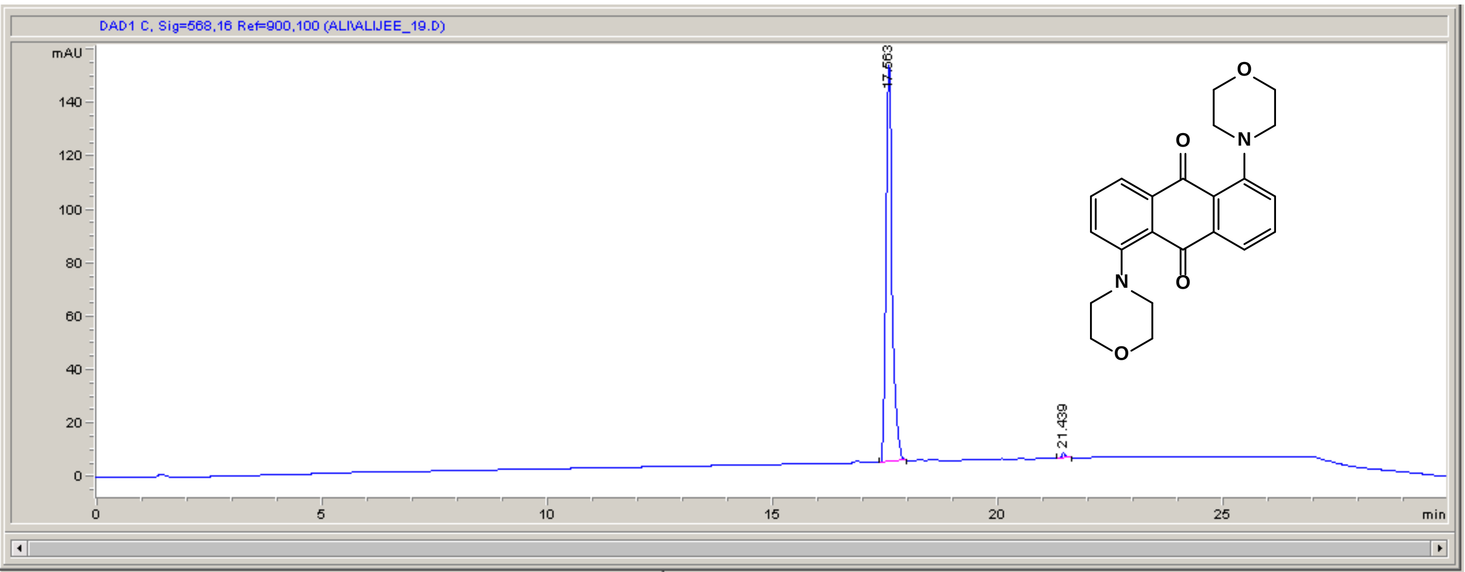

File Information

LC-File	ALIJEE_19.D
File Path	C:\CHEM32\1\DATA\ALI\
Date	17-Jan-16, 11:27:33
Sample	Jee9
Sample Info	
Barcode	
Operator	ali
Method	ALIAQ.M
Analysis Time	30 min
Sampling Rate	0.0067 min (0.402 sec), 4501 datapoints

#	Time	Area	Height	Width	Area%	Symmetry
1	17.563	1405.3	148.6	0.1403	99.069	0.679
2	21.439	13.2	1.8	0.1074	0.931	0.601

Compound 11

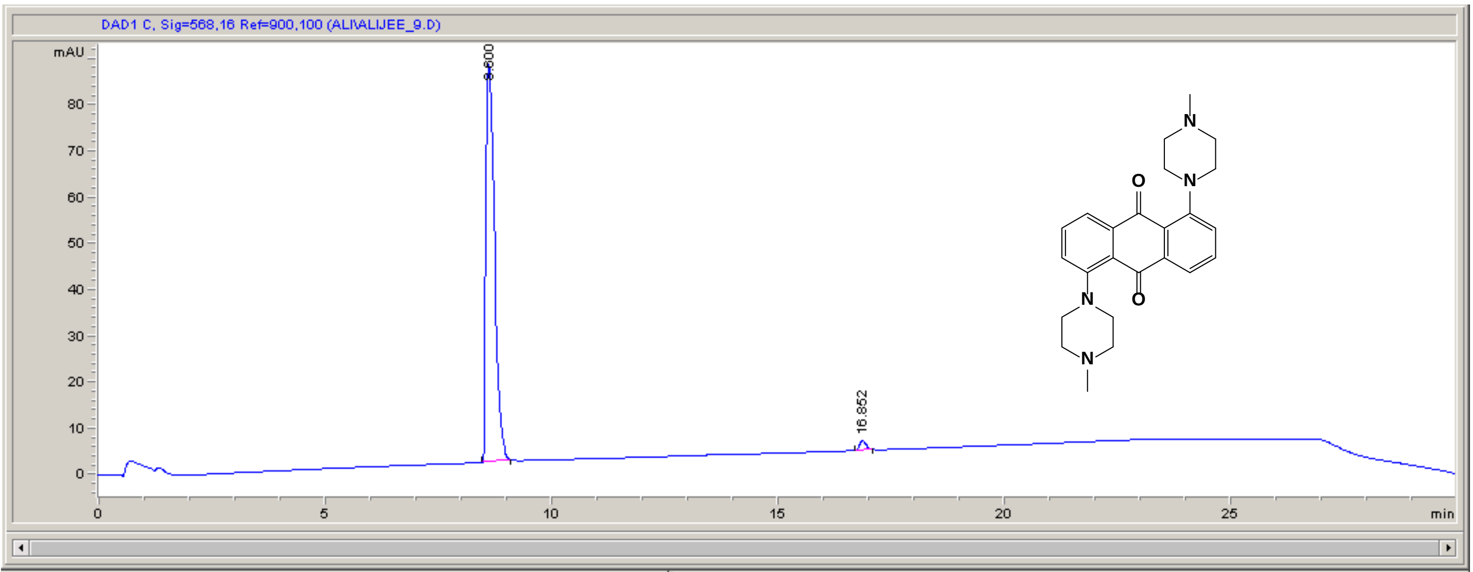

File Information

LC-File	ALIJEE_9.D
File Path	C:\CHEM32\1\DATA\ALI\
Date	16-Jan-16, 12:37:45
Sample	Jee6
Sample Info	
Barcode	
Operator	ali
Method	ALIAQ.M
Analysis Time	30 min
Sampling Rate	0.0067 min (0.402 sec), 4501 datapoints

#	Time	Area	Height	Width	Area%	Symmetry
1	8.6	1109	86.1	0.2042	98.138	0.451
2	16.852	21	2.1	0.1444	1.862	0.604

Compound 12

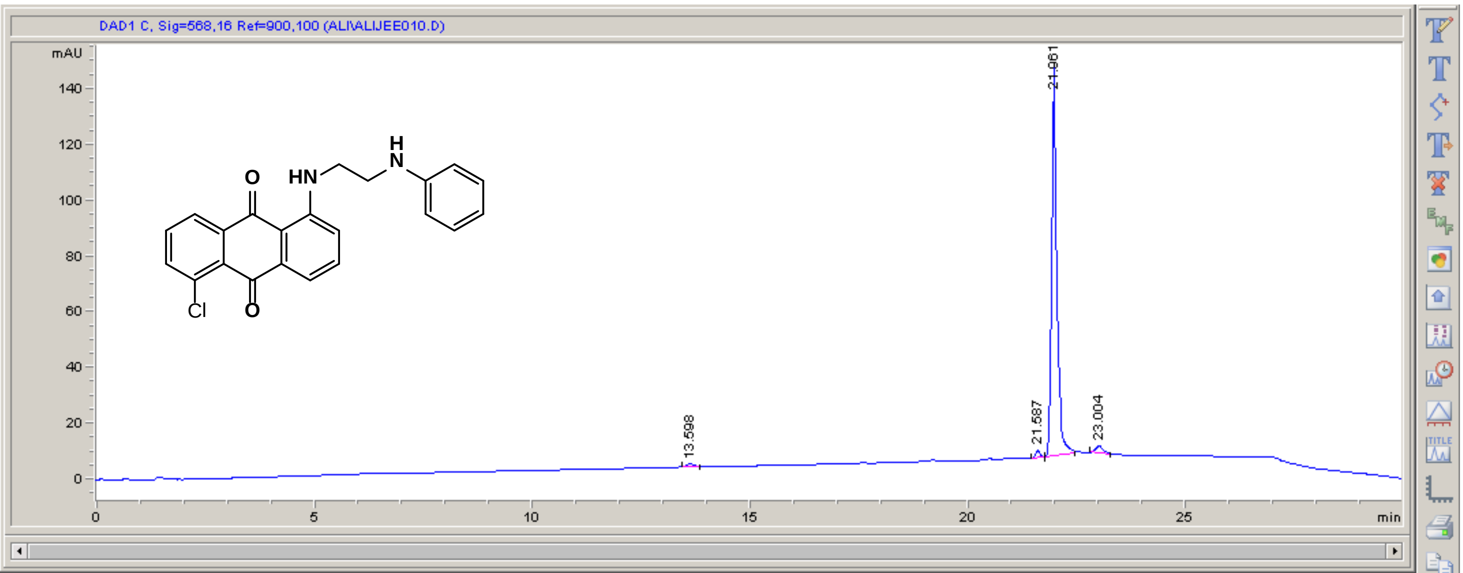

File Information

LC-File	ALIJEE010.D
File Path	C:\CHEM32\1\DATA\ALI\
Date	01-Feb-16, 12:27:23
Sample	JEE8 Pd
Sample Info	
Barcode	
Operator	ALI
Method	ALIAQ.M
Analysis Time	30 min
Sampling Rate	0.0067 min (0.402 sec), 4501 datapoints

#	Time	Area	Height	Width	Area%	Symmetry
1	13.598	12.4	1.1	0.167	0.976	0.633
2	21.587	21.3	2.8	0.1133	1.670	0.729
3	21.961	1208.3	140.4	0.1244	94.817	0.699
4	23.004	32.3	2.8	0.1688	2.537	0.838

Compound 13

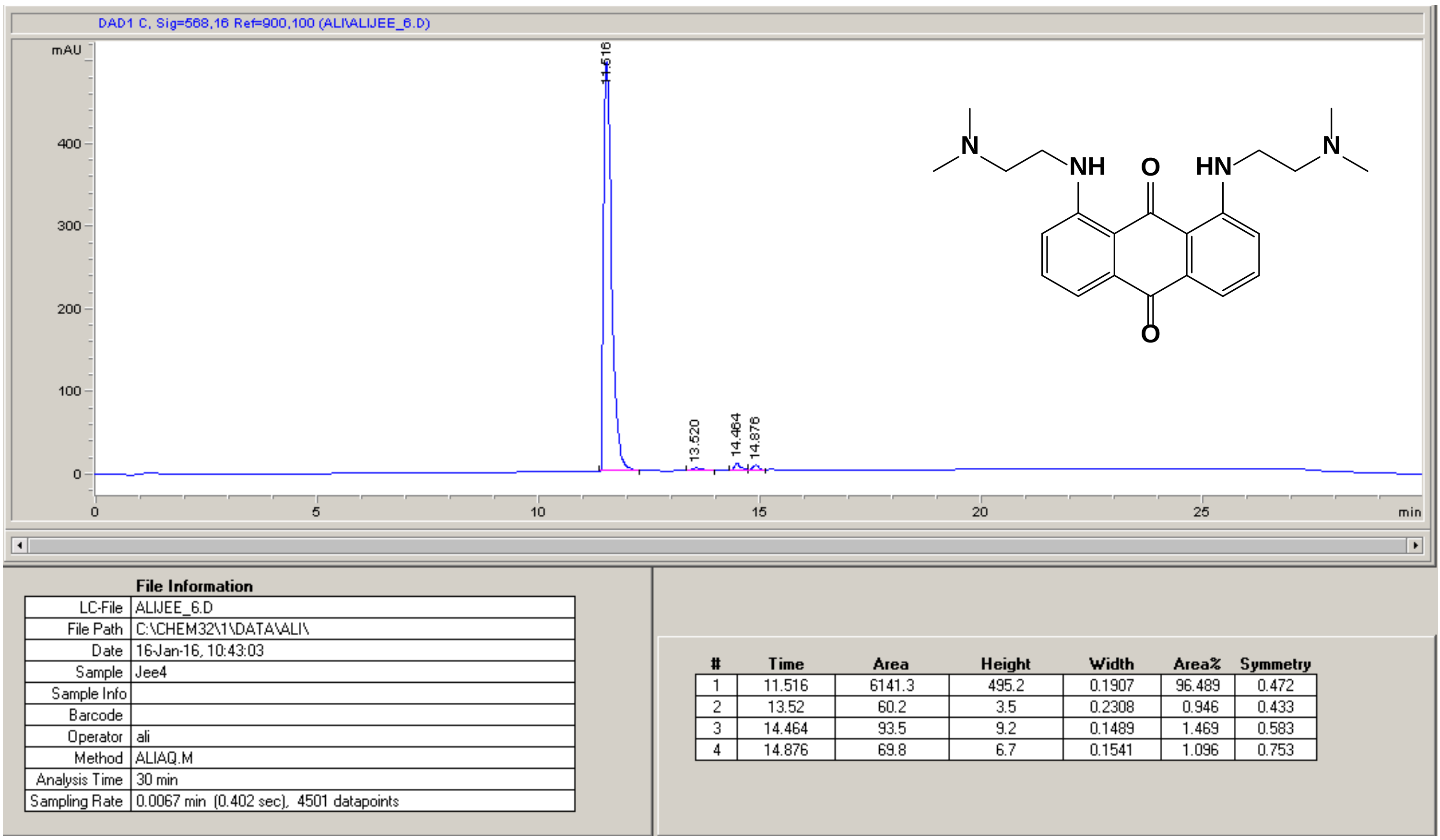

File Information

LC-File	ALIJEE_6.D
File Path	C:\CHEM32\1\DATA\ALI\
Date	16-Jan-16, 10:43:03
Sample	Jee4
Sample Info	
Barcode	
Operator	ali
Method	ALIAQ.M
Analysis Time	30 min
Sampling Rate	0.0067 min (0.402 sec), 4501 datapoints

#	Time	Area	Height	Width	Area%	Symmetry
1	11.516	6141.3	495.2	0.1907	96.489	0.472
2	13.52	60.2	3.5	0.2308	0.946	0.433
3	14.464	93.5	9.2	0.1489	1.469	0.583
4	14.876	69.8	6.7	0.1541	1.096	0.753

Compound 14

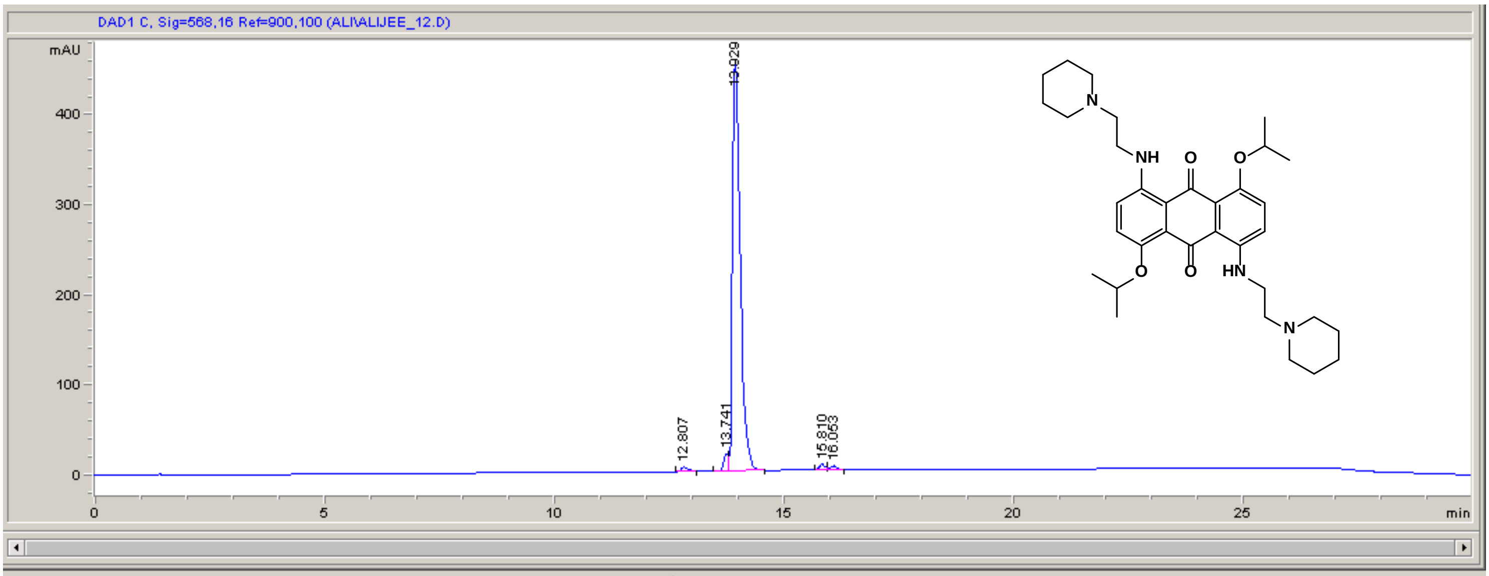

File Information

LC-File	ALIJEE_12.D
File Path	C:\CHEM32\1\DATA\ALI\
Date	16-Jan-16, 15:02:00
Sample	Jee16
Sample Info	
Barcode	
Operator	ali
Method	ALIAQ.M
Analysis Time	30 min
Sampling Rate	0.0067 min (0.402 sec), 4501 datapoints

#	Time	Area	Height	Width	Area%	Symmetry
1	12.807	47.6	4.7	0.1509	0.882	0.579
2	13.741	128.4	19.5	0.1007	2.381	1.77
3	13.929	5099	454.2	0.167	94.576	0.517
4	15.81	61.6	6.7	0.1346	1.143	0.75
5	16.053	54.9	4.7	0.1671	1.018	0.726

Compound 15

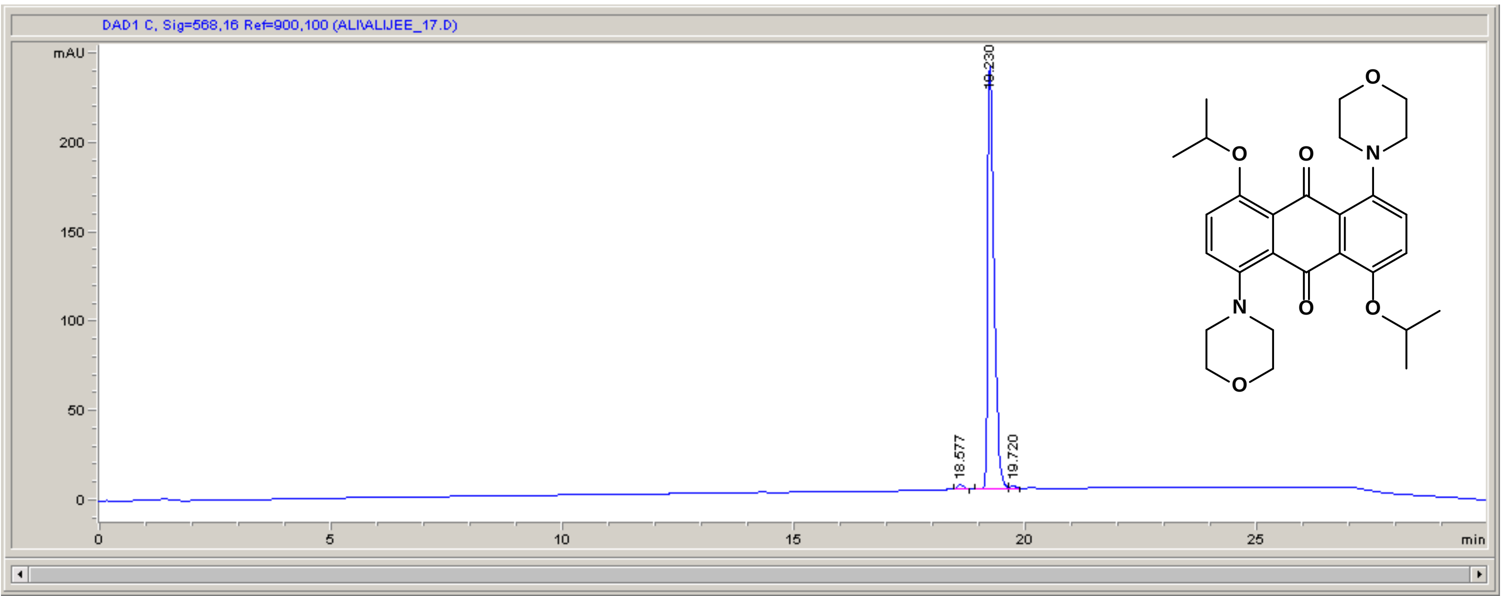

File Information

LC-File	ALIJEE_17.D
File Path	C:\CHEM32\1\DATA\ALI\
Date	17-Jan-16, 10:13:19
Sample	Jee12
Sample Info	
Barcode	
Operator	ali
Method	ALIAQ.M
Analysis Time	30 min
Sampling Rate	0.0067 min (0.402 sec), 4501 datapoints

#	Time	Area	Height	Width	Area%	Symmetry
1	18.577	27.3	2.8	0.1423	1.208	0.659
2	19.23	2218.5	236.8	0.1393	98.030	0.601
3	19.72	17.2	2.2	0.1108	0.761	0.697

Compound 16

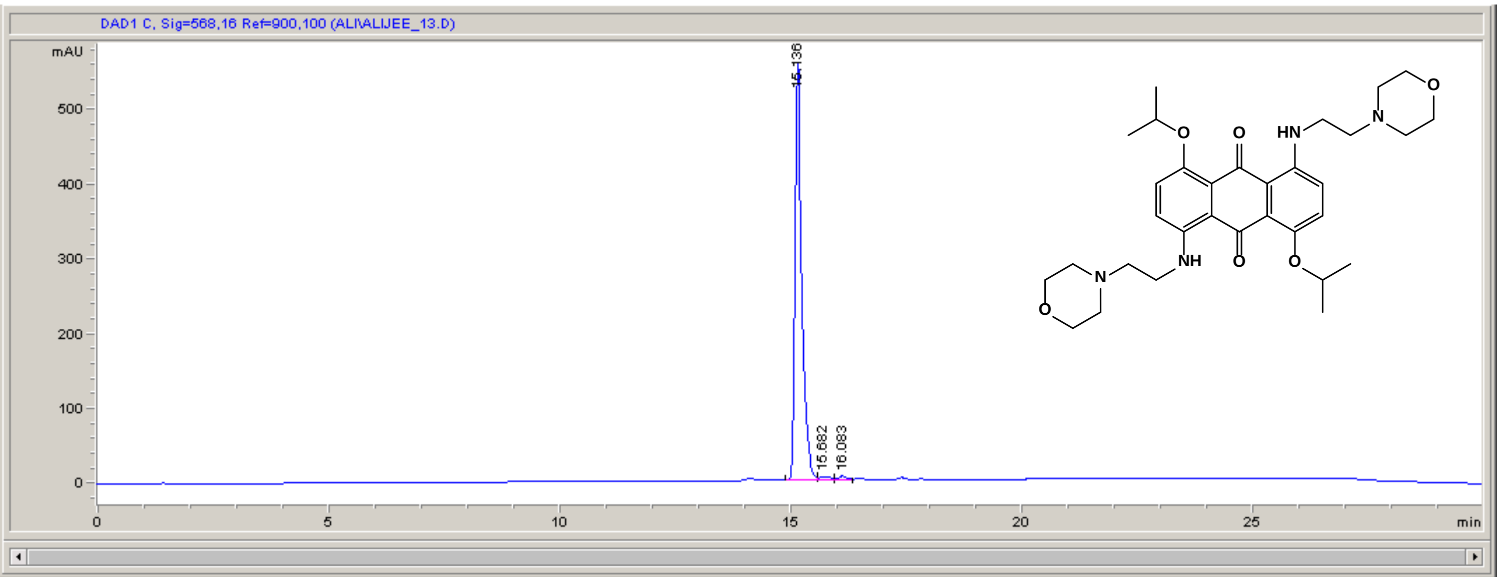

File Information

LC-File	ALIJEE_13.D
File Path	C:\CHEM32\1\DATA\ALI\
Date	16-Jan-16, 15:42:22
Sample	Jee18
Sample Info	
Barcode	
Operator	ali
Method	ALIAQ.M
Analysis Time	30 min
Sampling Rate	0.0067 min (0.402 sec), 4501 datapoints

#	Time	Area	Height	Width	Area%	Symmetry
1	15.136	5787.1	556.5	0.1553	97.587	0.569
2	15.682	80	4.6	0.2286	1.348	0.388
3	16.083	63.1	5.3	0.1701	1.065	0.615

Compound 17

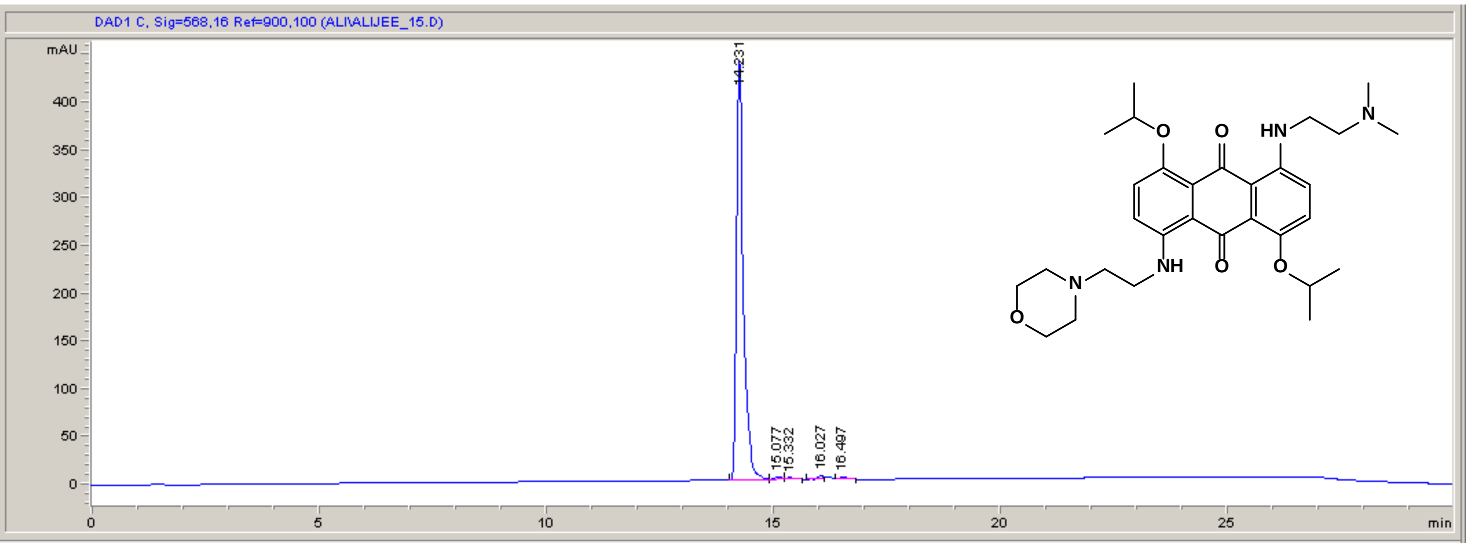

File Information

LC-File	ALIJEE_15.D
File Path	C:\CHEM32\1\DATA\ALI\
Date	17-Jan-16, 08:56:04
Sample	Jee22
Sample Info	
Barcode	
Operator	ali
Method	ALIAQ.M
Analysis Time	30 min
Sampling Rate	0.0067 min (0.402 sec), 4501 datapoints

#	Time	Area	Height	Width	Area%	Symmetry
1	14.231	4639.7	437.5	0.1577	96.797	0.527
2	15.077	40.5	3.5	0.1686	0.845	0.826
3	15.332	34.8	2.7	0.18	0.726	0.565
4	16.027	40.5	4.1	0.1423	0.845	1.234
5	16.497	37.7	3.2	0.1659	0.787	0.537

Compound 18

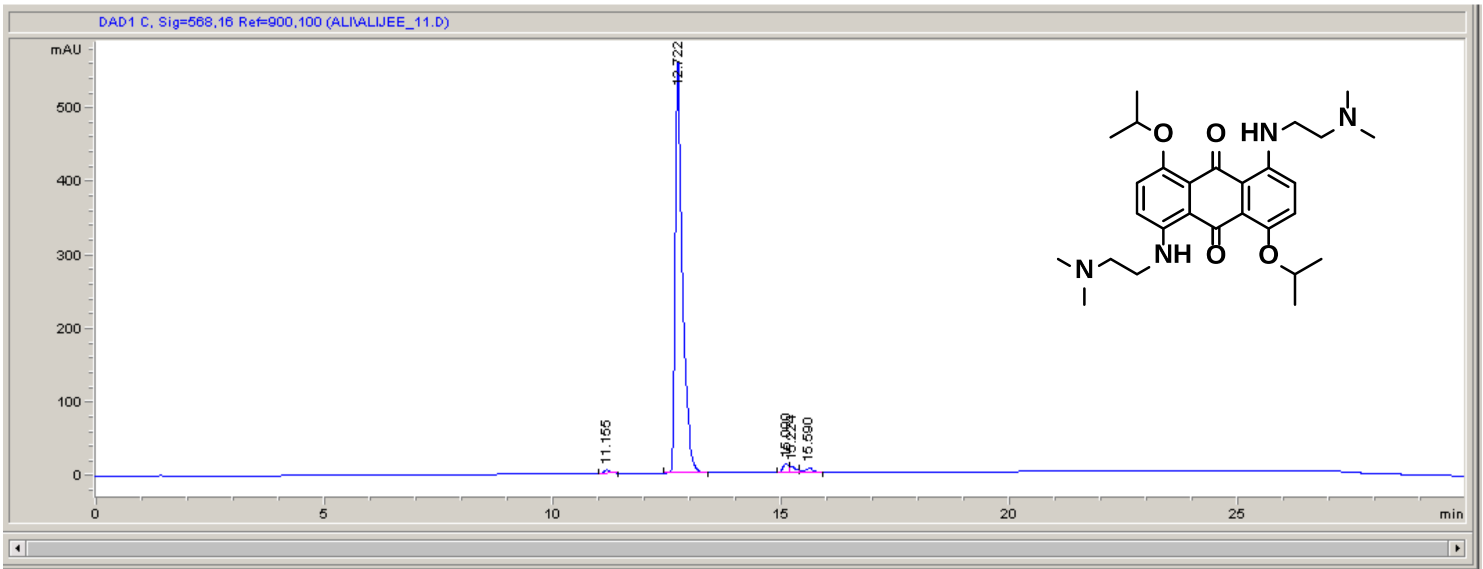

File Information

LC-File	ALIJEE_11.D
File Path	C:\CHEM32\1\DATA\ALI\
Date	16-Jan-16, 14:00:23
Sample	Jee11
Sample Info	
Barcode	
Operator	ali
Method	ALIAQ.M
Analysis Time	29.993 min
Sampling Rate	0.0067 min (0.402 sec), 4500 datapoints

#	Time	Area	Height	Width	Area%	Symmetry
1	11.155	51	5	0.1492	0.771	0.519
2	12.722	6307.6	558.8	0.1697	95.378	0.491
3	15.09	104.1	11.8	0.1311	1.575	0.933
4	15.224	70.8	8	0.1276	1.070	0.331
5	15.59	79.7	6.3	0.178	1.205	1.066

Compound 19

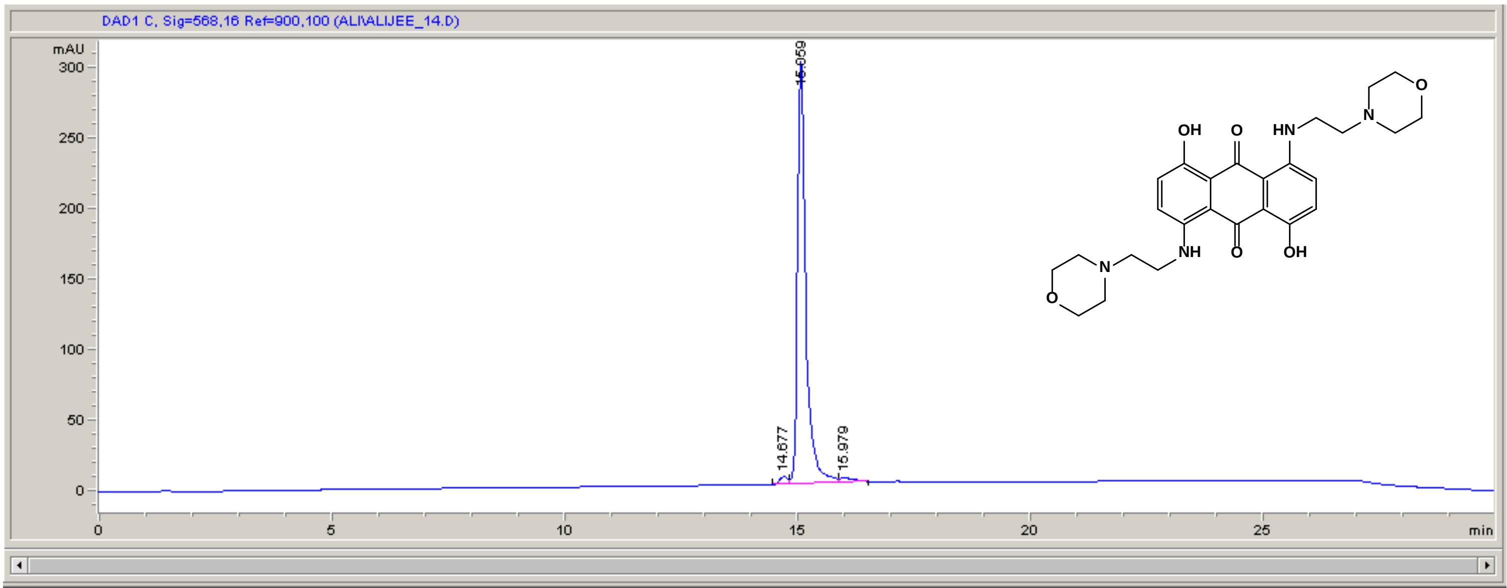

File Information

LC-File	ALIJEE_14.D
File Path	C:\CHEM32\1\DATA\ALI\
Date	16-Jan-16, 16:20:31
Sample	Jee18DP
Sample Info	
Barcode	
Operator	ali
Method	ALIAQ.M
Analysis Time	30 min
Sampling Rate	0.0067 min (0.402 sec), 4501 datapoints

#	Time	Area	Height	Width	Area%	Symmetry
1	14.677	62	5.1	0.1847	1.627	0.761
2	15.059	3673.1	299.1	0.1733	96.330	0.634
3	15.979	77.9	3.8	0.2644	2.043	0.334

Compound 20

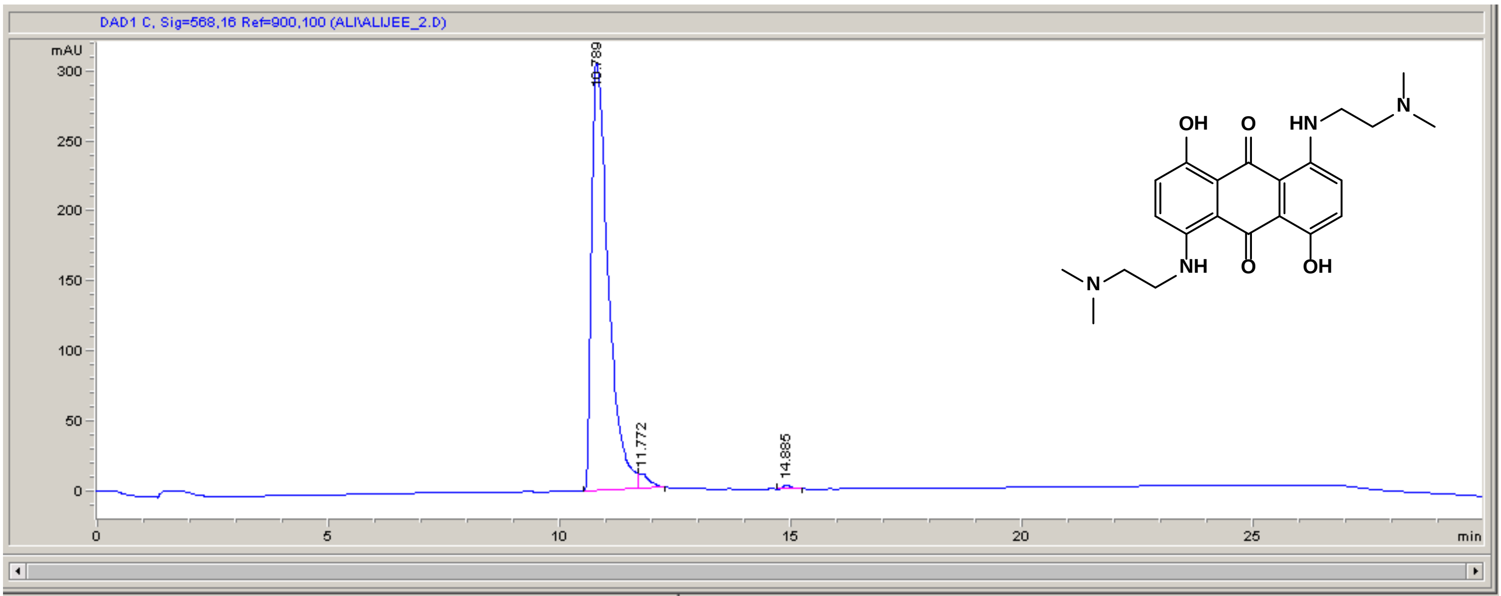

File Information

LC-File	ALIJEE_2.D
File Path	C:\CHEM32\1\DATA\ALI\
Date	15-Jan-16, 12:27:23
Sample	Jee11DP
Sample Info	
Barcode	
Operator	ali
Method	ALIAQ.M
Analysis Time	30 min
Sampling Rate	0.0067 min (0.402 sec), 4501 datapoints

#	Time	Area	Height	Width	Area%	Symmetry
1	10.789	7812.2	305.3	0.3885	97.367	0.429
2	11.772	174.7	10.9	0.2245	2.177	0.295
3	14.885	36.5	2.9	0.1811	0.455	0.474

8. SUPPORTING INFORMATION REFERENCES

1. Kypr, J., Kejnovska, I., Renciuk, D. & Vorlickova, M. Circular dichroism and conformational polymorphism of DNA. *Nucleic Acids Res.* **37**, 1713-1725 (2009).
2. Garbett, N.C., Ragazzon, P.A. & Chaires, J.B. Circular dichroism to determine binding mode and affinity of ligand-DNA interactions. *Nat. Protoc.* **2**, 3166-3172 (2007).
3. Abdallah, Q.M. et al. Minor structural modifications to alchemix influence mechanism of action and pharmacological activity. *Biochem. Pharmacol.* **83**, 1514-1522 (2012).
